# *In vitro* Studies of Transendothelial Migration for Biological and Drug Discovery

**DOI:** 10.3389/fmedt.2020.600616

**Published:** 2020-11-16

**Authors:** Alec T. Salminen, Zahra Allahyari, Shayan Gholizadeh, Molly C. McCloskey, Raquel Ajalik, Renee N. Cottle, Thomas R. Gaborski, James L. McGrath

**Affiliations:** ^1^Biomedical Engineering, University of Rochester, Rochester, NY, United States; ^2^Biomedical Engineering, Rochester Institute of Technology, Rochester, NY, United States; ^3^Bioengineering, Clemson University, Clemson, SC, United States

**Keywords:** transendothelial migration, extravasation, leukocytes, metastasis, *in vitro* platforms, drug discovery, hepatocyte transplantation, porous membranes

## Abstract

Inflammatory diseases and cancer metastases lack concrete pharmaceuticals for their effective treatment despite great strides in advancing our understanding of disease progression. One feature of these disease pathogeneses that remains to be fully explored, both biologically and pharmaceutically, is the passage of cancer and immune cells from the blood to the underlying tissue in the process of extravasation. Regardless of migratory cell type, all steps in extravasation involve molecular interactions that serve as a rich landscape of targets for pharmaceutical inhibition or promotion. Transendothelial migration (TEM), or the migration of the cell through the vascular endothelium, is a particularly promising area of interest as it constitutes the final and most involved step in the extravasation cascade. While *in vivo* models of cancer metastasis and inflammatory diseases have contributed to our current understanding of TEM, the knowledge surrounding this phenomenon would be significantly lacking without the use of *in vitro* platforms. In addition to the ease of use, low cost, and high controllability, *in vitro* platforms permit the use of human cell lines to represent certain features of disease pathology better, as seen in the clinic. These benefits over traditional pre-clinical models for efficacy and toxicity testing are especially important in the modern pursuit of novel drug candidates. Here, we review the cellular and molecular events involved in leukocyte and cancer cell extravasation, with a keen focus on TEM, as discovered by seminal and progressive *in vitro* platforms. *In vitro* studies of TEM, specifically, showcase the great experimental progress at the lab bench and highlight the historical success of *in vitro* platforms for biological discovery. This success shows the potential for applying these platforms for pharmaceutical compound screening. In addition to immune and cancer cell TEM, we discuss the promise of hepatocyte transplantation, a process in which systemically delivered hepatocytes must transmigrate across the liver sinusoidal endothelium to successfully engraft and restore liver function. Lastly, we concisely summarize the evolving field of porous membranes for the study of TEM.

## Introduction

The vascular system exists as a connection between all tissues and organs. In addition to a plethora of soluble molecules, many cell types are critically circulated by blood flow throughout the body. With both remedial and pathological outcomes, immune, and metastatic cancer cells use the circulatory system to navigate to sites of interest. Similarly, stem cells systemically administered for therapeutic applications freely travel within circulation until a target tissue is reached (1). While the mechanisms and reasons for cell dissemination and extravasation may differ in these scenarios, one inevitable step in each case is the crossing of the vascular endothelial barrier, also known as transendothelial migration (TEM).

*In vivo*, the extravasation cascade is a complex process involving both changes in protein expression and the structure of the vascular endothelium. Immune and cancer cells alike may alter protein expression or affinity to aid in the capture, rolling, firm adhesion, and diapedesis (TEM) of the cells out of circulation (2, 3). This cascade is a well-studied and highly characterized process, particularly for immune leukocytes, that has benefitted greatly from the use of *in vitro* model systems. Leaving the realm of traditional murine studies, *in vitro* systems provide a highly controlled platform for experimentation. Additionally, the reductionism afforded by these platforms permits meticulous characterization of the mechanisms that facilitate each step of the extravasation cascade.

Despite significant advances in pharmacology, drug discovery remains an inherently costly task. Furthermore, low clinical trial success rates (4) highlight the poor predictive nature of animal models for testing of efficacy and safety. The immune response in mice, for example, is known to be markedly different than humans when given a similar systemic inflammatory insult (5). These findings help explain the failure of promising therapies in clinical trials, such as those aimed at treating the devastating inflammatory disease, sepsis (6).

One promising avenue to rekindle the stagnant pre-clinical drug discovery approach is the use of sophisticated and highly controllable *in vitro* platforms. For both a variety of inflammatory diseases and cancers, *in vitro* platforms that permit the study of leukocyte or cancer cell transmigration may help elucidate novel drug targets promoting beneficial, or limiting damaging, cell extravasation. In this review, we will provide an overview of the current understanding of immune and cancer cell extravasation, with a keen focus on TEM, as strengthened by both traditional and modern microphysiological systems. Furthermore, we will highlight particularly promising areas of research for novel anti-inflammatory and anti-metastatic drug development. Lastly, we will comment on emerging biological and engineering topics concerning the field of TEM.

## Immune Cell Extravasation

**Figure 1:**
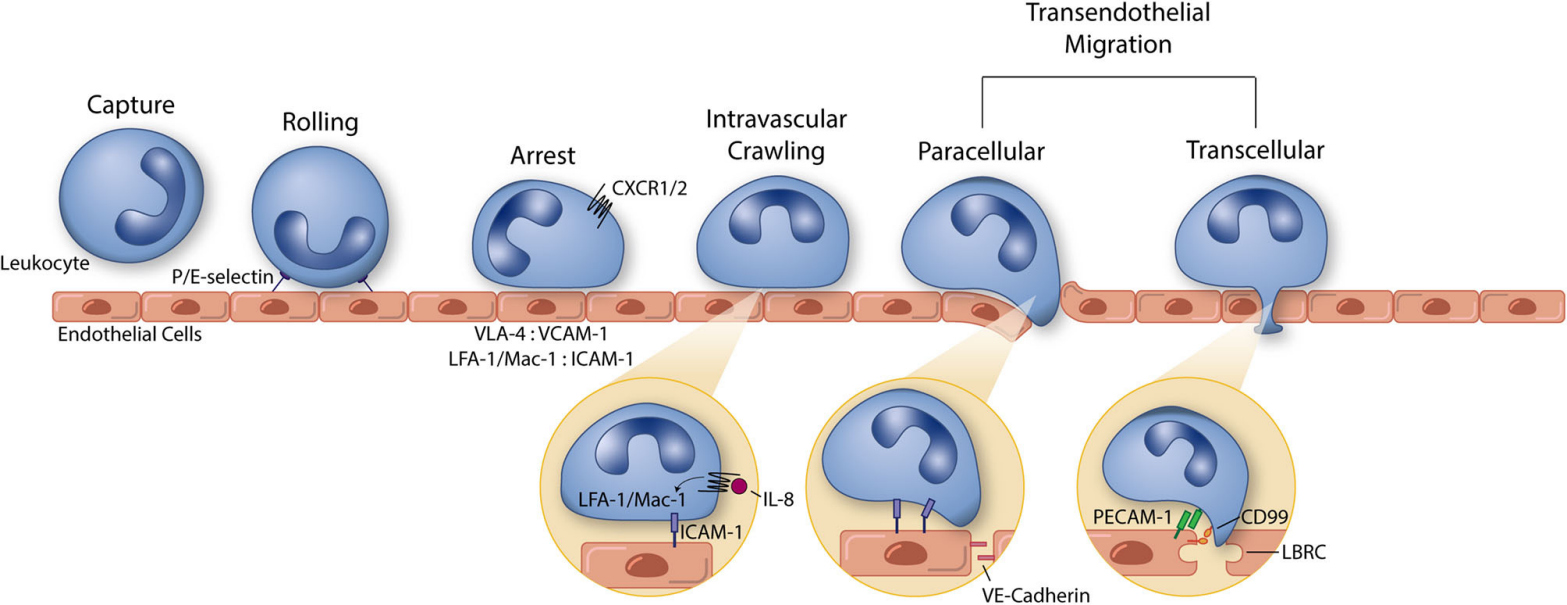
The leukocyte extravasation cascade is an elaborate process understood in part due to the use of well-controlled *in vitro* studies. Platforms incorporating vascular endothelial cells and physiological environmental conditions (e.g., shear stress) permit the highly controlled study of all steps of the leukocyte extravasation cascade: capture, rolling, arrest, intravascular crawling, and both paracellular and transcellular transendothelial migration. P and E selectin bind leukocytes to aid in the capture and rolling phases of extravasation. LFA-1/Mac-1: ICAM-1 and VLA-4: VCAM-1 form high affinity/avidity integrin/ligand interactions to halt leukocytes on the apical endothelial cell surface during arrest. Integrin activation is aided by chemokine signaling (neutrophil chemokine IL-8 and its receptors CXCR1/2 pictured here). In addition to leukocyte arrest and intravascular crawling, LFA-1/Mac-1: ICAM-1 interactions function to signal VE-cadherin junctional turnover and opening of the endothelial cell-cell junctions. Additionally, PECAM-1 and CD99 homophilic interactions between leukocytes and endothelial cells function to drive membrane mobilization from the lateral border recycling compartment (LBRC) to increase membrane surface area around the transmigrating leukocyte. The understanding of each of these components of leukocyte extravasation have been guided by the use of simple and modern *in vitro* systems. These same devices can, in turn, function as early stage drug discovery platforms for preventing devastating inflammatory diseases.

Recognition and clearance of pathogens is a key function of leukocytes in immunity. While innate and adaptive leukocytes have distinct roles in the immune response to foreign bodies, one conserved feature of nearly all leukocytes is the ability to traverse the vascular endothelium. As such, leukocyte extravasation (Figure 1) involves a tightly regulated cascade of signaling and mechanical events that end in the TEM of leukocytes to the tissue. While this phenomenon is essential in mounting a remedial response to bacteria or viruses, dysregulated immune cell extravasation underlies the pathogenesis of many human diseases, including rheumatoid arthritis, Crohn's disease, multiple sclerosis, psoriasis, atherosclerosis, and sepsis (7, 8). Therefore, improving our understanding of molecular mechanisms of leukocyte extravasation, as well as quickly screening pharmaceuticals aimed at limiting dysregulated TEM specifically, will greatly impact the clinic. In this section, we will review the use of *in vitro* mimetics for the elucidation of leukocyte extravasation mechanisms (summarized in Table 1) and avenues for drug discovery.

**Table 1:** Summary of highlighted *in vitro* studies on immune cell transendothelial migration.

**References**	**Endothelium**	**Migratory cell line**	**Extracellular matrix**	**Inflammatory stimulus**	**Flow**
Allingham et al. (9)	Human umbilical vein	HL-60 & primary human neutrophils	Matrigel	10 ng/mL TNF-α	
Bixel et al. (10)	Mouse bend.5	Mouse neutrophils	Laminin	5 nM TNF-α or 10 ng/mL IL-1β	
Buffone et al. (11)	Human umbilical vein	HL-60 & primary human neutrophils		IL-1β	100 to 800 s^−1^
Carman et al. (12)	Human umbilical vein	Primary human neutrophils, monocytes, & lymphocytes	Fibronectin	100 ng/mL TNF-α	
Green et al. (13)	Transfected L-cells, CHO, or human umbilical vein	Primary human neutrophils		200 U/mL TNF-α	1 dyn/cm^2^
Khire et al. (14)	Human umbilical vein	Primary human neutrophils	Collagen I gel & fibronectin		10 dyn/cm^2^
Mossu et al. (15)	Human brain like	T cells	Gelatin	10 ng/mL TNF-α	0.1–1.5 dyn/cm^2^
O'Brien et al. (16)	REN mesothelioma	Primary human neutrophils	Fibronectin	10 U/mL IL-1β or 100 U/mL TNF-α	
Salminen et al. (17)	Human umbilical vein	Primary human neutrophils	Collagen I gel or fibronectin		4.5 dyn/cm^2^
Shaw et al. (18)	Human umbilical vein	Primary human neutrophils	Fibronectin	25 ng/mL TNF-α	1 dyn/cm^2^
Steiner et al. (19)	Primary mouse brain microvessel	T cell line SJL.PLP7	Matrigel	25 ng/mL TNF-α	0.25–1.5 dyn/cm^2^
Watson et al. (20)	Human umbilical vein or mouse heart	Human mononuclear leukocytes & neutrophils or mouse leukocytes	Collagen I gel	TNF-α or IL-1β	
Yang et al. (21)	Human umbilical vein	Primary human neutrophils & T cells	Fibronectin	25 ng/mL TNF-α	1 dyn/cm^2^

### Capture/Rolling

The first step in the leukocyte extravasation cascade is the recruitment and adhesion of immune cells to the apical endothelial cell (EC) surface. A major barrier to this process is the shear force exerted on circulating leukocytes preventing non-specific adhesion to the vessel walls. Immune cells can overcome these shear forces and adhere to the endothelium via a variety of molecular interactions, most prominently selectins. While the molecules involved in leukocyte adhesion vary by cell and tissue, there is much overlap in mechanisms, and thus can be described in general terms.

Adhesion is initiated by low-affinity binding and rolling across the endothelium. With high on and off rates, these interactions can occur under flow, and sometimes even require flow (2). In addition, these interactions keep cells in close contact with the endothelium to permit the initiation of the subsequent step in the extravasation cascade, leukocyte arrest. Leukocytes bind to varying selectins and cell adhesion molecules depending on immune cell class. These molecules and their respective ligands/receptors are upregulated by ECs and immune cells when exposed to pro-inflammatory cytokines, such as tumor necrosis factor alpha (TNF-α) and Interleukin 1 beta (IL-1ß) (22–25). Cytokines can also induce degradation of the EC glycocalyx to increase exposure of these molecules on the apical EC surface for adhesion (26). Further, degradation of the glycocalyx can in turn induce a greater endothelial response (27). Which molecules are upregulated and to what extent they are upregulated depends on the stimulus that cells are exposed to and the concentration of stimulus (24, 28). In addition, binding can occur in a tissue-dependent manner due to inherent differences of ECs in different tissues [i.e., EC heterogeneity (29)]. Given these complexities, *in vitro* systems have permitted highly controlled studies that focus on specific immune cells and tissues to understand diverse extravasation mechanisms.

Both *in vitro* and *in vivo* studies have shown that primary adhesion and rolling of neutrophils and monocytes occur mainly via three selectins: E-selectin, P-selectin, and L-selectin, and their ligands (2, 22, 30). Details about these selectins have been reviewed extensively elsewhere (31, 32). In addition to selectins, integrins play a role in slowing down rolling cells to allow for firm adhesion (2, 30). While lymphocytes can bind selectins during primary adhesion and rolling, they may also roll along intercellular adhesion molecule 1 (ICAM-1) and vascular cell adhesion molecule 1 (VCAM-1) (2, 23, 33). The role of cell adhesion molecules in high-affinity binding following rolling but prior to diapedesis will be discussed in greater detail later in this review.

Several *in vitro* flow systems have been utilized to study rolling and capture of immune cells. Because these steps occur prior to transmigration, most studies have utilized well-established flow systems, such as the circular parallel plate flow chamber, the flow chamber system, and perfusion flow loop/straight tube models (27, 33–35). Most of these systems utilize syringe pumps or cyclic motion to analyze rolling and adhesion under flow conditions. While simple, these systems have been instrumental in establishing the roles of specific selectins and other molecules. For example, Green and colleagues demonstrated that endothelial E-selectin induces clustering and colocalization of L-selectin and PSGL-1 on neutrophil microvilli during rolling using a flow chamber system. This redistribution is critical for activation and firm adhesion of the leukocyte, and the *in vitro* flow system mimicking the shear stress of the post-capillary venules [1 dyn/cm^2^, a primary location of leukocyte diapedesis (36)] allowed the mechanisms of redistribution and activation to be discovered (13). More sophisticated models have also been developed in recent years to better mimic *in vivo* conditions. For example, Benson et al. developed a microfluidic device using photolithography that models the scale and mechanics of *in vivo* post-capillary venules to study the effects of extensional stress and capillary extension on leukocyte adhesion (37). Initially, in the absence of red blood cells (RBC), leukocytes (freshly-isolated peripheral blood mononuclear cells, PBMCs) were captured at initial extensional regions due to formation of aggregates and secondary capture. This is not physiologically relevant, however, so the addition of RBCs was crucial in this model. After RBC addition, slow rolling across an ICAM-1-coated surface was observed, highlighting the platforms potential use to understand mechanisms of rolling and capture. Further, Prabhakarpandian et al. were able to create microfluidic networks via digitization of *in vivo* microvessels and soft-lithography. By modeling the complex geometry more accurately than accomplished by the parallel plate flow chamber, they were able to observe primary adhesion of particles (anti-P-selectin conjugated) largely at bifurcations of branches (P-selectin coated), which is in agreement with *in vivo* data (38).

It is important to note that while the studies presented to this point demonstrate the impact of shear flow on leukocyte capture, ECs alone are also greatly influenced by flow. For example, Santaguida et al. monitored bovine aortic endothelial bed permeability, as measured by transendothelial electrical resistance (TEER), under static and flow conditions. A shear stress of 4 dyn/cm^2^ (mimicking the peak shear of the post-capillary venules) had a profound impact on peak TEER, with dynamic cells reaching a peak resistance of ~65 Ohm cm^2^ vs. 10 Ohm cm^2^ in static cultures (39). Voyvodic et al. built and characterized a custom flow chamber to further characterize the effects of shear on human umbilical vein endothelial cells (HUVECs). Under a shear stress of 20 dyn/cm^2^ [A shear level representative of flow in the capillaries (36)], HUVECs were observed to elongate and align in the direction of flow (40). This elongation was observed more readily in HUVECs sheared with laminar/steady vs. pulsatile flow, demonstrating the importance of flow characterization in these platforms. Together, these studies urge the consideration of flow priming of ECs in vascular models. However, the added complexity of advanced systems like these typically comes with the cost of reducing manufacturability and throughput. Given that these platform goals are particularly important in drug discovery, as compound screening must be performed in well-controlled and high-volume platforms (41), studies must pre-consider the importance of EC elongation and barrier enhancement before designing around these challenging outcomes. Nonetheless, *in vitro* systems incorporating shear stress modules have greatly contributed to our understanding of leukocyte capture and exist as an important tool for drug discovery.

### Firm Adhesion, Arrest, and Luminal Crawling

Following selectin mediated leukocyte capture and rolling, leukocyte integrins engage EC counter receptors to achieve firm adhesion and arrest (42). The process of immune cell arrest involves a series of adhesive events including affinity, valency, and binding stabilization, all combining to control the overall avidity or adhesiveness of the immune cell-substrate interaction (43).

To initiate cell arrest, immune cell expressed G-protein coupled receptors (GPCRs) bind chemokines, signaling to induce changes in integrin binding affinity (inside-out signaling). GPCR inside-out signaling functions to alter integrin structure; in an inactivated state, integrins hold a bent low-affinity position that transitions to an extended, high-affinity conformation during the course of activation (44). The extended high-affinity conformation exposes the ligand binding pocket of the target integrins, allowing for firm adhesion to the apical EC surface. The most well-characterized integrins involved in this process are β_2_ integrins, leukocyte function-associated antigen 1 (LFA-1; α_L_β_2_ or CD11a-CD18) and macrophage-1 antigen (Mac-1; α_M_β_2_ or CD11b-CD18) (45). Both LFA-1 and Mac-1 bind the EC-expressed ligand, ICAM-1 (CD54). ICAM-1 is basally expressed in low levels on the cell membrane of ECs, however, inflammation is associated with an increase in ICAM-1 expression and presentation, allowing for increased LFA-1 and Mac-1 binding, ultimately driving immune cell arrest (19, 21).

While many chemokines are hypothesized to be involved in immune cell arrest [relevant review (46)], one chemokine of particular importance in vascular inflammation is the C-X-C chemokine interleukin 8 (IL-8; CXCL8) (47–49). IL-8 is produced by ECs (as well as fibroblasts, macrophages, and mast cells) and signals through immune cell expressed GPCRs, CXCR1, and CXCR2. Notably, murine IL-8 homologs (KC, MIP-1, and LIX) do not accurately reflect the function of IL-8 in humans (50), therefore, traditional murine models are not suitable for the study of IL-8 without exogenous perfusion. As a result, many early studies of IL-8 effects on leukocyte recruitment and arrest were performed *in vitro*, using human cells.

In a study by DiVietro and colleagues, a laminar flow chamber was utilized to probe the effects of IL-8 on CD18 integrin mediated ICAM-1 binding (51). *In vivo*, IL-8 is presented on the apical/luminal EC surface in an insoluble (proteoglycan-bound) form (52). Therefore, this study examined the effects of IL-8 immobilized to a firm surface. Primary human neutrophils were perfused over a polystyrene surface coated with ICAM-1 (50 sites/μm^2^), IL-8 (350 sites/μm^2^), or a coimmobilization of the two. In so doing, they found a sharp increase in neutrophil arrest when perfused over the combined coating as compared to either coating individually. When neutrophils were again perfused over the combined coating in the presence of TS1/18 (anti-CD18 IgG1), cell arrest returned to the baseline observed on the ICAM-1 only surface. This study highlights the ability of IL-8 to activate β_2_ integrins in the process of immune cell arrest. Similar studies utilizing parallel plate flow chambers, with the addition of a HUVEC monolayer, demonstrated the importance of IL-8 in driving the arrest of monocytes (53) and eosinophils (54). Both innate immune cell types were more prone to arrest on inflamed HUVECs in the presence of IL-8. While antibody blocking of β_2_ integrins again had a negative effect on monocyte arrest in the presence of IL-8, less effect was seen in the eosinophil population. Given the aforementioned disconnect between murine and human inflammation, these *in vitro* studies were ultimately critical in advancing the understanding of IL-8 in immunity, particularly cell arrest. Since the discovery and characterization of IL-8, multiple other chemokines (e.g., CXCL6, CCL2, and CXCL12) have been elucidated for their key roles in inflammation and homeostatic immune function [reviewed in depth here (55)]. Secretion of these chemokines by ECs and tissue resident immune cells establish complex chemotactic gradients, driving inflammation resolution or adaptive immune surveillance. *In vitro* platforms can further help us understand how a microenvironment of competing chemokines [e.g., host-derived IL-8 vs. pathogen-derived N-formylmethionine-leucyl-phenylalanine (fMLP)] may resolve (56), and determine what chemokines are most adept for drug targeting.

Once bound to the apical EC surface, immune cells begin to traverse the cell membrane in pursuit of a location for diapedesis. This process of intravascular crawling involves specific regulation of integrin binding and cell polarization, allowing immune cells to efficiently navigate to a site of vascular exit (2). While advances in *in vivo* imaging techniques have greatly contributed to the understanding of immune cell intravascular crawling, the benefits of the high spatial and temporal resolution afforded by *in vitro* vascular mimetics is just beginning to be realized (14, 17).

Similar to immune cell arrest, regulation of LFA-1 and Mac-1 binding to ICAM-1 plays a major role in defining immune cell crawling. The distinct functions of these molecules in neutrophil crawling was investigated in a study by Buffone et al. HUVECs were seed on tissue-culture polystyrene plates, exposed to 10 ng/mL of recombinant IL-1β (>4 h), and assembled into a parallel-plate flow chamber (11). To determine the migration patterns of leukocytes on the inflamed endothelium, two neutrophil sources were used: (1) the human leukemia cell, neutrophil-like HL-60s and (2) freshly isolated primary human neutrophils. While not primarily derived, HL-60s have an added benefit over freshly isolated neutrophils as they may be cultured indefinitely in their pre-differentiated state, adding great flexibility to study design (57, 58). In either case, neutrophils preferentially migrated with the direction of flow on stimulated HUVEC monolayers in an integrin dependent manner. When LFA-1 was blocked, any previously observed upstream migration was completely attenuated. Interestingly, when Mac-1 was blocked, migration became preferentially upstream (against the direction of flow), suggesting key and divergent roles of LFA-1 and Mac-1 in determining the direction of neutrophil crawling on the apical surface of inflamed ECs. While upstream crawling is not typical of wild-type/untreated innate immune cells *in vivo*, native Th1 memory/effector T cells are known to preferentially crawl against flow on brain microvessels *in vivo* (59). This phenomenon was reconstituted *in vitro* on TNF-α stimulated primary mouse brain microvascular endothelial cells (pMBMECs) (19). CD4^+^ Th1 memory/effector T cells showed preferential migration against flow on inflamed pMBMECs grown in a parallel flow chamber. In agreement with Buffone et al. LFA-1 and ICAM-1 were determined to be critical in regulating this phenomenon. Given Th1 memory/effector T cells do not express Mac-1, the ability of these cells to migrate upstream in the brain microvascular *in vivo* may be attributed to the sole expression of LFA-1.

In addition to ICAM-1, LFA-1 and Mac-1 utilize ICAM-2 for immune cell arrest and crawling (60, 61), however, the adhesive strength of this bond is far less than the respective integrin bond to ICAM-1 (62), thus the role it plays in these phenomena is less well-understood. Furthermore, Steiner et al. ruled out ICAM-2 as a possible mediator of upstream migration of Th1 memory/effector T cells *in vitro* (19). T cells crawling on wild-type primary mouse brain microvascular endothelial cells (pMBMECs) primarily migrated against the direction of flow. While ICAM-1 knockout in pMBMECs had an inhibitory effect on tracked upstream migration, ICAM-2 knockout had no effect. Overall, these studies support the conclusion that intravascular crawling of both innate and adaptive immune cells is not random and is instead regulated in a manor to provide quick resolution of the insulting target. Whether therapeutics targeting intravascular crawling may prove beneficial to the clinic remains to be determined.

### Transendothelial Migration/Diapedesis

Diapedesis, the step in which a migrating cell moves from the luminal to the abluminal sides of the vascular wall, is known to occur by two distinct mechanisms: paracellular (between the endothelial cell-cell contacts) and transcellular (through the EC body). These different routes of TEM have been observed *in vitro* utilizing high-resolution light microscopy. In a study by Carman and Springer, TNF-α stimulated [100 ng/mL for 12 h] HUVECs (plated on fibronectin coated glass) were exposed to chemoattractants specific to monocytes, neutrophils, or lymphocytes, rinsed, and the respective leukocytes were added (63). Slides were transferred to an incubation chamber atop a high-resolution confocal microscope and images were taken at 100X to distinguish instances of paracellular vs. transcellular migration. For all three leukocyte subgroups, ~90% of TEM took the paracellular route. The authors note, however, that some instances of migration appeared to resemble transcellular diapedesis, but were too close to the cell-cell junction to distinguish from paracellular migration. We suspect this may be a common feature of transcellular migration as the thinnest part of the EC, and thus the most energetically favorable location for diapedesis, tends to be at the periphery. Furthermore, there is growing evidence that EC heterogeneity and inflammatory environment variation may regulate the decision to emigrate para- vs. transcellularly. In a study by Carman et al. lymphocyte transcellular migration was observed in 10% of total diapedesis events across HUVECs *in vitro* (12). Under the same conditions, microvascular cell lines permitted ~30% transcellular migration. Thus, heterogeneity between macro and microvascular ECs may play a role in facilitating para- vs. transcellular leukocyte TEM. Additionally, Yang et al. discovered that neutrophil transcellular migration was dependent on ICAM-1 function, density, and distribution; HUVECs expressing high levels of ICAM-1 favored neutrophil transcellular migration, which was largely attenuated by ICAM-1 disruption (21). Inflammatory environments that induce greater levels of ICAM-1 expression may, in turn, favor transcellular leukocyte TEM. The use of super-resolution microscopy (STED/STORM) and compatible *in vitro* platforms may help better elucidate the exact percentage of cells that transmigrate through the cell body. Nonetheless, *in vitro* studies have cemented the concepts of trans- and paracellular migration, urging future work to explore the exact molecular mechanisms underlying each method.

While a complete understanding of the molecular basis of leukocyte TEM is lacking, *in vitro* models have greatly advanced our understanding of proteins key in these processes. One well-characterized player in the process of TEM is the immunoglobulin superfamily protein, platelet endothelial cell adhesion molecule −1 (PECAM-1, CD31). PECAM-1 is natively expressed at high levels at the endothelial cell-cell junctions, as well as on a number of leukocytes, including monocytes, neutrophils, and subclasses of T cells (64). PECAM-1 signals through homophilic, PECAM-1-PECAM-1 interactions, hence the importance of both vascular and immune cell expression (65). An early *in vitro* study utilizing anti-PECAM-1 blocking antibodies and a simple leukocyte transmigration assay assessed the reliance of PECAM-1 in the process of monocyte TEM (66). Primary human monocyte TEM was quantified on TNF-α stimulated [10 ng/mL] HUVEC monolayers (seeded on hydrated collagen gels in a 96-well plate to permit complete monocyte TEM) in the presence of anti-PECAM-1 monoclonal Ab hec7 or isotype-matched control Ab. Nearly 100% TEM was reported in the control monocyte group compared to <20% TEM in the presence of the anti-PECAM-1 Ab (regardless of whether monocytes were pre-incubated or Ab was added at time of assay). This work established PECAM-1 as a key component of leukocyte TEM in the presence of inflammatory cytokines. Interestingly, 20% of monocytes remain capable of TEM, thus PECAM-1 blocking is not sufficient to completely halt leukocyte TEM under these conditions.

The effects of PECAM-1 on leukocyte TEM *in vitro* was also assessed using the well-established Costar Transwell™ platform (16). This work established the following: (1) PECAM-1 is not upregulated on ECs (as measured by immunofluorescence) following inflammatory cytokine (IL-1β or TNF-α) stimulation, (2) PECAM-1 blocking (via anti-PECAM-1 mAb) does not alter leukocyte adhesion or luminal crawling upstream of diapedesis, and (3) Leukocyte TEM involves both PECAM-1 dependent and independent mechanisms. This was evident in the case of chemokine driven TEM, as PECAM-1 blocking had no effect on IL-8 or leukotriene B4 (LTB_4_) induced neutrophil TEM. A complementary *in vitro* study utilizing high-resolution immunofluorescence to observe PECAM-1 and EC membrane dynamics concluded what we now believe to be the main function of PECAM-1 in TEM: PECAM-1 signaling mobilizes membrane from a cellular region known as the lateral border recycling compartment (LBRC) (67) to the site of leukocyte TEM, increasing EC membrane surface area and surrounding the immune cell in the intermediate stages of diapedesis (68). Thus, PECAM-1 signaling has been established as a facilitating (but not essential) factor in leukocyte TEM using *in vitro* platforms.

Downstream of PECAM-1-PECAM-1 signaling is a second set of homophilic interactions between leukocyte and EC expressed CD99 (10). Much like PECAM-1, CD99 is primarily localized at the EC border, and plays a distinct role in leukocyte diapedesis (69). Unraveling the specific function of CD99 in leukocyte TEM has been greatly aided by the use of *in vitro* transmigration assays. In a study by Watson et al. anti-CD99 monoclonal Abs drastically reduced monocyte transmigration across TNF-α activated HUVEC monolayers (grown on hydrated type I collagen gels) as compared to a non-blocking anti-vascular endothelial (VE)-cadherin control Abs (20). Further experimentation demonstrated that CD99 (constitutively expressed in a complex with ezrin) engagement activates soluble adenylate cyclase to locally produce cAMP and activate protein kinase A, ultimately directing a second wave of LBRC targeting to the site of TEM. CD99 signaling was also determined to be critical in neutrophil transmigration across inflamed (IL-1β stimulated) HUVEC monolayers *in vitro*: Anti-CD99 blocking resulted in an 80% reduction in TEM as compared to the anti-VE-cadherin non-blocking control (70). Thus, PECAM-1 and CD99 function in similar but separate and sequential manners to direct EC membrane mobilization from the LBRC to the site of TEM, allowing for proper leukocyte TEM. Interestingly, even though PECAM-1 and CD99 tend to localize to the EC junction, the role of LBRC recycling appears to be conserved across both trans- and paracellular TEM. The exact molecular mechanisms within the LBRC underlying TEM post-LBRC recycling is less clear and remains to be studied. While PECAM-1 and CD99 expression is not necessarily altered in inflammation, these molecules clearly play a role in the immune response in inflammation and exist as potential high yield therapeutic targets for limiting over-excessive leukocyte TEM. Importantly, however, ubiquitous expression of PECAM-1 and CD99 limits the ability to specifically target regions of inflammation if inhibitors are delivered systemically. Developing therapeutic vectors that target regions of inflammation and subsequently inhibit PECAM-1 and CD99 function on a local level are critical for the success of these potential therapeutics if off-target immunosuppressive effects are to be avoided.

The final, well-understood molecular mediator of leukocyte TEM is VE-cadherin (cadherin-5, CD144). VE-cadherin is an adherens junction protein and major component of the vascular endothelial barrier and thus a barrier to transmigrating leukocytes (71). Unlike PECAM-1 and CD99, VE-cadherin's role in leukocyte transmigration is exclusive to the paracellular route. Of note, VE-cadherin junctions are disrupted in inflammation even in the absence of leukocytes. Whether vascular barrier dysfunction in inflammation is exclusively a result of leukocyte-independent mechanisms or a combination of leukocyte-dependent and independent mechanisms is often debated [reviewed previously (72)], however, it is clear VE-cadherin rearrangement does have a major role in facilitating paracellular leukocyte TEM. Observational studies utilizing optically favorable *in vitro* vascular models were critical in establishing the participation of VE-cadherin in TEM. In a study by Shaw et al. HUVECs were plated on fibronectin-coated glass coverslips and subsequently transduced with a VEcadGFP adenoviral vector to allow for real-time observation of VE-cadherin dynamics without directly inhibiting the function of the adherens junction (18). Transduced HUVECs were grown to confluency and stimulated with TNF-α [25 ng/mL for 4–6 h]. Primary human neutrophils and monocytes were perfused over the HUVEC surface and transmigration was observed under sequential fluorescence and differential interference contrast (DIC) imaging. The authors note a definitive and transient opening of VE-cadherin cell-cell junctions at sites of TEM. However, some instances of leukocyte TEM took place at sites of pre-existing VE-cadherin gaps, particularly at multicellular junctions (3 or more cells). In the case of VE-cadherin opening, the authors report quick resealing (~5 min) of the junction post-TEM, thus this process is quick and non-permanent as to not disrupt the vascular barrier for prolonged periods of time.

Recently, our lab studied the effects of chemokine driven neutrophil TEM on vascular barrier integrity using a novel vascular mimetic (14). While most TEM studies employ the use of inflammatory cytokines, or a combination of cytokines and chemokines, our work aimed to directly probe the consequence of neutrophil transmigration in the absence of EC inflammatory effects. This work was aided by the use of an optically transparent nanoporous membrane that allowed for fMLP presentation to the basal channel of the two-channel vascular mimetic. We ultimately observed dynamic opening and closing of HUVEC-expressed VE-cadherin junctions at sites of TEM that was coupled with a drop in transendothelial impedance (representative of barrier integrity), reflecting the ability of leukocytes to disrupt HUVEC junctions in the absence of inflammatory cytokines. These changes in barrier integrity, however, are minor, and may not contribute greatly to the overall effects of inflammation, particularly in cases of few TEM events.

Following seminal observational studies like that of Shaw et al. researchers have begun to identify upstream mediators of VE-cadherin gap formation in leukocyte TEM. Tyrosines Y658 and Y731 on the cytoplasmic tail of VE-cadherin bind p120 and either β-catenin or plakoglobin, respectively, to link VE-cadherin to the actin cytoskeleton (71). Phosphorylation of these tyrosine residues, potentially a downstream result of ICAM-1 or VCAM-1 signaling, dissociates these bonds, allowing for VE-cadherin endocytosis and weakening of the cell-cell junction (73). This mechanism was found critical for the facilitation of neutrophil TEM *in vitro*: blocking Y685 or Y731 phosphorylation on VE-cadherin (within HUVECs seeded on a Transwell™ filter) significantly decreased primary human neutrophil TEM as compared to control (9). A more recent *in vitro* TEM study by Gonzalez and colleagues elucidated that this phosphorylation event is downstream of LBRC targeting, as selective blocking of LBRC recycling resulted in a loss of VE-cadherin gap formation and decreased leukocyte TEM (67). Conversely, inhibiting Y658 and Y731 phosphorylation did not alter LBRC targeting. Together, these studies highlight the critical role of VE-cadherin modulation in facilitating successful leukocyte TEM.

## Metastasis

Whereas, immune cell TEM can pertain to remedial or maladaptive actions, the intravasation or extravasation of carcinoma cells is exclusively pathogenic. Cancer cell dissemination (i.e., metastasis) accounts for 90% of disease mortality (74), and thus exists as a potentially high yield target for the treatment of carcinomas. These pharmaceuticals belong to a class of drugs aptly termed migrastatics, juxtaposing the more well-understood class of cancer drugs, cytostatics (anti-cancer cell proliferation) (75). Like immune cell extravasation, the low cost, high throughput, and optimal imaging permitted by *in vitro* systems has greatly influenced our understanding of metastasis (summarized in Table 2) and can open the door to a future of effective drug discovery platforms in pursuit of novel cancer therapeutics.

**Table 2:** Summary of highlighted *in vitro* studies on cancer cell transendothelial migration.

**References**	**Endothelium**	**Migratory cell line**	**Extracellular matrix**	**Inflammatory stimulus**	**Flow**
Chen et al. (76)	Human umbilical vein	MDA-MB-231, A-375, MA2, & 4T1	Fibrin/Collagen I gel		5 dyn/cm^2^
Cui et al. (77)	Primary human vascular	MDA-MB-231 & MCF-10A	Poly-D-lysine & fibronectin		2.5–10 dyn/cm^2^
Hamilla et al. (74)	Human umbilical vein	MDA-MB-231, A375, & SW1990	Fibronectin	25 ng/mL TNF-α	
Herman et al. (78)	Human cerebral microvessel or primary rat brain	A2058, B16/F10, MDA-MB-231, & 4T1	Rat tail collagen		
Jeon et al. (79)	Human microvascular	MDA-MB-231 & MCF-10A	Collage I gel & matrigel		
Li et al. (80)	Bovine aortic	Calu-1, HT-1080, Colo 205, HT-29, SW 620, SW 480, & L-132	Matrigel		
Ni et al. (81)	Human pulmonary microvessel	A459	Collagen I gel		0.05 dyn/cm^2^
Song et al. (82)	Human dermal microvascular	MDA-MB-231	Fibronectin	50 ng/mL TNF-α	0.5–2.5 dyn/cm^2^
Zervantonakis et al. (83)	Human microvessel & umbilical vein	HT1080 & MDA-MB-231	3D ECM (non-specific)	2 ng/mL TNF-α	

### Epithelial-Mesenchymal Transition

**Figure 2:**
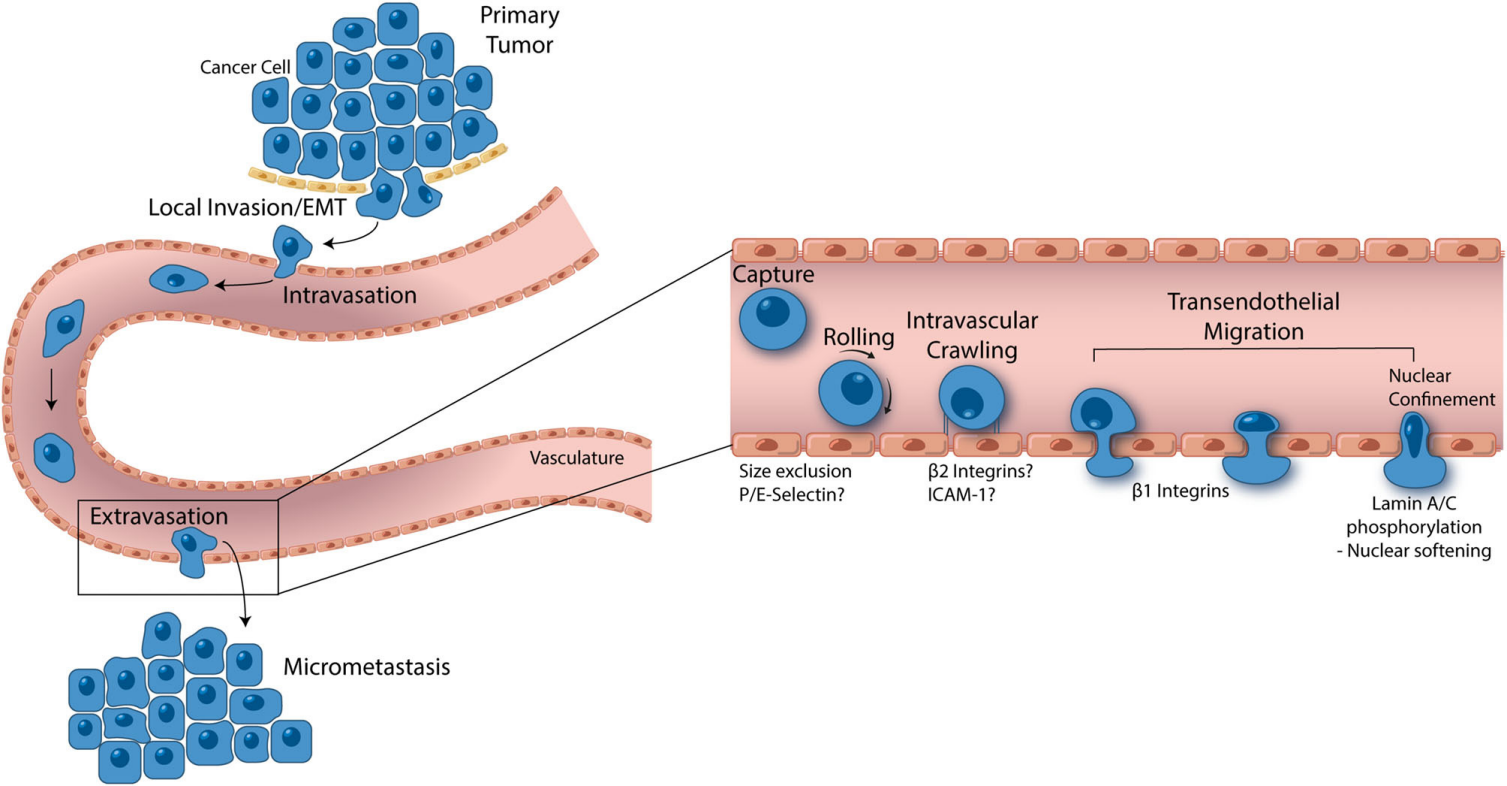
From local invasion to micrometastasis formation, metastatic cancer cells take a complex, and strenuous path to complete the metastasis cascade. While the percentage of cancer cells able to successfully form a secondary tumor is low, metastasis accounts for 90% of cancer mortality, thus making it one of the most important oncological processes that can be exploited for drug discovery. Cancer cells begin the metastasis cascade by transitioning from a static, epithelial phenotype to a dynamic, mesenchymal phenotype (EMT), and invading surrounding tissue. Once on the basal endothelial cell surface, metastatic cancer cells may intravasate into circulation, moving across the endothelium from the abluminal to luminal surface. Shear blood flow releases the cancer cell from the luminal vascular wall where it is carried to a secondary location. Size restriction in the capillaries halts the cancer cell, where β_1_ integrins can attach and facilitate transendothelial migration. In some cases, P and E selectin as well as β_2_ integrins have been implicated in cancer cell extravasation, however, more studies are needed to understand the role these proteins play. Given the ridged nature of the cell nucleus, all contents of a transmigrating cancer cell are pushed into the basement membrane until the last minute when which the nucleus is squeezed through. Lamin A/C phosphorylation permits nuclear softening and severe deformation, aiding in the push across the endothelium. Successful transendothelial migration constitutes the final step in forming a secondary tumor. *In vitro* models have greatly contributed to these fresh understandings of metastasis and are opening the door to novel drug targets.

The metastasis cascade (Figure 2) begins with the transition of cancer cells from an adherent, cobblestone, epithelial morphology, into a spindle-shaped, mesenchymal morphology in a process termed epithelial-mesenchymal transition (EMT) (84). While not all cancers involve EMT, it is likely that the metastasis of nearly all carcinomas, a class of cancers that makes up 80–90% of all cancers, involves EMT (85). During EMT, cancer cells begin to invade the normal tissue parenchyma surrounding the tumor. Eventually tumor cells locate and intravasate the vascular endothelium in an abluminal to luminal manor, permitting dissemination through circulation. With 90% of cancer related deaths associated with metastasis (86), EMT, invasion, and intravasation are of high interest in the clinical setting.

EMT involves a cascade of signaling events and transcription factors leading to distinct morphological and protein changes characterizing the transition. This process confers resistance to many immune and chemotherapeutics (87), and thus represents a key focus for effective treatment of ensuing carcinoma metastasis/progression. Additionally, the ability of these cancer cells to migrate to and through the vascular endothelium must be achieved for metastasis to occur. Ultimately, only 0.01% of circulating tumor cells lead to the formation of metastatic tumors at secondary sites (88). Developing therapeutics that block or limit cancer cell EMT, invasion and intravasation will greatly reduce the metastatic potential of carcinomas.

EMT is largely regulated by transcription factors (EMT-TFs) that suppress or induce genes favoring the cell transition from an epithelial state to a mesenchymal state. The most well-characterized transcription factors involved in EMT include the zinc-finger E-box binding homeobox factors ZEB1 and ZEB2, SNAIL, SLUG, and the basic helix-loop-helix factors TWIST1 and TWIST2 (89). EMT is often initiated by various environmental factors (e.g., hypoxia) (90), growth factors (e.g., TGF-β) (91), and extracellular matrix proteins. Following induction of EMT, EMT-TFs repress epithelial proteins that regulated apical and basal membrane distinction, cell-cell adhesion, and cell-basement membrane adhesion: Adherens junction protein E-cadherin, tight junction proteins occludins and claudins, and α_6_β_4_ integrins are all repressed in EMT. Additionally, these same transcription factors function to induce genes favoring the mesenchymal phenotype: N-cadherin, vimentin, fibronectin, matrix metalloproteinases (MMPs), and β_1_ and β_3_ integrins are all upregulated in EMT. How these factors regulate certain steps in the transition cascade and how they differ depending on cancer origin, has been researched in-depth [reviewed here (84)]. There are many intermediate phenotypes that determine the invasive potential of cells undergoing the EMT transition (92), thus the process is complex and sporadic to confer metastatic potential.

While EMT has been studied on simple 2D culture substrates, recent work has demonstrated the importance of recapitulating the complex 3D microenvironment to properly model EMT *in vitro*, potentially opening doors to advanced drug screening and cancer biology tools. In a study by Pal et al. hybrid scaffolds were designed to mimic the fibrous extracellular matrix for physiological tumor growth (93). Poly Lactic-co-Glycolic Acid (PLGA) was electrospun to form porous scaffolds that were then coated with gelatin methacrylamide (GelMA) to provide adhesive contacts for physiological cell growth. The final composite contained a biochemical microenvironment provided by the GelMA with filamentous structures provided by the electrospun PLGA. MDA-MB-231 breast cancer cells exhibited elevated expression of EMT-TFs SNAIL, ZEB1, and TWIST2 when grown in the composite scaffold as compared to PLGA or GelMA alone. Similarly, MKN74 gastric cancer cells had significantly higher expression of SNAIL and ZEB1 compared to scaffold components alone. Thus, this work demonstrated the importance in replicating the biochemical, dimensional, and architectural features of the *in vivo* tumor microenvironment when studying EMT *in vitro*. As our ability to readily build microphysiological systems advances, platforms like that of Pal et al. will greatly aid in the ability to explore potential EMT-associated molecular targets for cancer treatment, if supplemented with traditional chemotherapies to reduce initial tumor presence.

The study of EMT-conferred chemotherapy resistance may also be aided by the use of microphysiological systems. Ki et al. demonstrated the ability of pancreatic ductal adenocarcinoma (PDAC) cells to resist treatment with the chemotherapy drug gemcitabine when cultured in 3D hydrogels as compared to standard 2D cultures, potentially due to the post-EMT phenotype of the cells seeded in 3D microenvironments (94). Therefore, microphysiological systems may also provide key insight into cancer-specific drug resistance and directly inform clinical treatment.

### Invasion and Intravasation

Microphysiological tools have also been developed to study the invasion of cancer cells into the surrounding extracellular matrix and intravasation across the vascular endothelium into circulation. These devices include both an extracellular matrix component as well as regions for the co-culture of vascular ECs. In a study by Zervantonakis et al. a microfluidic device consisting of three parallel microchannels was constructed for the systematic study of cancer cell invasion and intravasation (83). The channel configuration allowed for co-culture of fibrosarcoma HT1080 or breast carcinoma MDA-MB-231 cancer cell lines with primary microvascular endothelial cells (MVECs) or HUVECs. Tumor and endothelial cells were seeded in distinct channels and were separated by a 500 μm wide extracellular matrix hydrogel. *In situ* live cell imaging permitted the study of cancer cell intravasation across normal and perturbed (TNF-α stimulated) EC barriers. Zervantonakis et al. ultimately observed elevated tumor cell intravasation across perturbed EC barriers, highlighting the potential role of inflammation in this early step of metastasis. Similar devices have been used by others for the controlled study of invasion and intravasation at the lab bench (95–97). Plug-and-play microfluidic devices have also been developed, permitting independent study of each step in the invasion-metastasis cascade. One such device featured in the work by Ni and colleagues includes a removable U-well insert that permits tumor cell, extracellular matrix, and EC additions to study EMT, invasion, intravasation, and cell detachment into circulation under flow (81). Given the importance of cancer cell EMT, invasion, and intravasation on metastasis, tools like these will ultimately guide future drug development for the prophylactic treatment of cancer metastasis that, if successful, could limit the mortality of most cancers (98).

### Capture

The mechanisms involved in cancer cell capture (the beginning of the cancer cell extravasation cascade) on the apical EC surface are still not fully understood. It is possible that many features of this process are relatively conserved across immune and cancer cells, as studies have observed both cell classes utilizing selectins and integrins during rolling and primary adhesion. Unlike leukocytes, however, which express selectins themselves, cancer cells express only the ligands for selectins found on the endothelium (31, 99), limiting their function in capture.

Using flow cells similar to those used for leukocyte studies, investigations have begun to unravel the key molecules and mechanisms involved in metastatic cell capture on the vascular wall. Several studies have elucidated a key role for E-selectin and its various ligands (99–102). Shea et al. utilized micropatterning of E-selectin and HA to elucidate their roles in pancreatic cell rolling and adhesion. They discovered that E-selectin promotes adhesion of pancreatic cancer cells to HA and arrest of cells on the endothelium by both slowing the cells and bringing them in close proximity to the surface (101). Edwards et al. used photoconvertible protein technologies to study single cell velocities of colon carcinoma cells during rolling and adhesion. The microfluidic device was functionalized with selectins at tunable ratios for a reductionist approach to elucidate the roles of each selectin (103). Additionally, Spencer and Baker developed a cone and plate device within a 96-well plate format, which, combined with electric cell-substrate impedance sensing, can analyze cancer cell adhesion to the extracellular matrix or cell monolayers under flow conditions in a high throughput manner. They elucidated differences between two breast cancer cell lines (MDA-MB-231 and MCF-7). For MDA-MB-231, adhesion to a collagen II-coated surface increased under flow conditions, whereas it decreased for MCF-7. The high throughput nature of this devices allows these differences to be discovered more readily (104). These studies suggest potential parallels between the capture of immune cells and cancer cells by endothelium but also provide methods to elucidate important differences for the purposes of drug targeting.

### Arrest and Luminal Crawling

β_1_ integrins have been hypothesized to play a role in cancer cell arrest *in vivo* (105) and *in vitro* (106). β_1_ integrins primarily bind EC basement membrane components (as well as the EC expressed ligand, VCAM-1) (107). Therefore, while immune cells preferentially bind with β_2_ integrins to proteins upregulated in inflammation, cancer cells may arrest in uninflamed environments, further distinguishing the targeted and sporadic nature of immune and cancer cell TEM, respectively. A study by Song et al. however, suggests that inflammation may still promote cancer cell arrest (82). In this work, a custom microfluidic device was designed that allowed for cytokine stimulation of a human dermal microvascular endothelial cell (HDMEC) monolayer in one location and no stimulation elsewhere in the same device. In so doing, they observed more cancer cell adhesion in cytokine activated ECs compared to the non-cytokine exposed endothelium. Still, other studies point to a non-molecular, size restriction mechanism for cancer cell arrest. Given the larger size of cancer cells compared to immune cells, they are often forced into contact with the walls of narrowed microvasculature (108).

Once arrested on the apical EC surface, cancer cell may exhibit migratory behavior. *In vivo* studies have highlighted intravascular crawling of cancer cells (109, 110), however, this area of research has not been carefully examined in vascular mimetics. While some studies claim to observe intravascular crawling (77, 111), quantification and/or molecular mechanisms were not explored. We suspect this is due to the relatively static nature of cancer cells in relation to the highly motile immune cells. This is evident in a study by Bapu et al. in which extended time-lapse microscopy was utilized to observe cancer cell intravascular crawling (112). Representative images depict cancer cells migrating roughly 20–50 μm over 4 h. Thus, while the cells appear to crawl, the speed associated with this migratory behavior is minimal, requiring lengthy studies. Despite this difficulty, investigating cancer cell migration on vascular ECs will greatly benefit from the imaging and quantification afforded by *in vitro* platforms and should prove beneficial in the pursuit of novel migrastatics.

### Transendothelial Migration

Cancer cell crossing of the vascular endothelium in the process of TEM has long been considered a key step in metastasis (113). However, many of the mechanisms involved in this process are only now being elucidated. This is further complicated by the fact that cancer cells exist in many forms, therefore the proteins involved in diapedesis of certain classes of cancer cells may not match those of others. Nonetheless, *in vitro* devices have allowed for direct observation of single cell transmigration in models of metastasis, laying the foundation to better understand the mechanisms involved.

Early *in vitro* studies of cancer cell TEM utilized simple Boyden chamber configurations to probe the ability of cancer cell lines to cross the endothelium. In a study by Li and Zhu, seven tumor cell lines (Calu-1, HT-1080, SW 620, SW 480, HT-29, L-132, and Colo 205) were analyzed for their ability to traverse a bovine aortic endothelial cell (BAEC) monolayer atop a Transwell™ filter (80). Radioactivity of Cr-51-labeled, transmigrated tumor cell lines were quantified to assess the degree of cell TEM. While this study (and many other studies utilizing Boyden chambers) was limited in imaging capability, the radiometric technique highlighted the variability in cancer cell TEM potential across the seven tested tumor cell lines. The authors conclude that variations in observed EC retraction resulting from tumor cell-release cytotoxic or enzymatic factors may be responsible for the subsequent TEM variability. Lastly, this work also established the extended time needed for complete cancer cell TEM (>4 h).

More recent work takes advantage of the optical clarity afforded by *in vitro* devices to better understand the processes involved in cancer cell TEM. Like leukocytes, cancer cells have been shown to traverse the endothelium both through the paracellular and transcellular routes (78). Cancer cells may also incorporate into the endothelium during TEM in a process distinct from paracellular leukocyte TEM: MDA-MB-231, A375, and SW1990 cancer cell lines were observed incorporating into HUVEC monolayers *in vitro* (74). The process involved EC rounding and dispersal as the cancer cell pushed into and spread across the underlying basement membrane. The total process lasted anywhere from 15 to 60 min, representative of a relatively quick method of transmigration. Overall, however, the most well-studied mechanism of cancer cell TEM is β_1_ integrin-dependent paracellular migration (114). In a study by Chen et al. a custom design microfluidic device was used to observe MDA-MB-231 cancer cells breaching a HUVEC monolayer (76). The process of TEM begins with a thin protrusion through the HUVEC cell-cell junction to the underlying basement membrane. Over the course of 3–4 h, the basement membrane side protrusion begins to grow, all while maintaining a minimal gap in the endothelium (~1 μm). At the last minute, the ridged cancer cell nucleus is deformed [a key mechanism in cancer cell TEM (115)] and pushed through a widening cell-cell junction (~ 8 μm), a process that lasts ~15 min and constitutes the final act of TEM. β_1_ integrin knock down (through stable expression of shRNA targeting integrin β_1_) in MDA-MB-231 cancer cells prevented protrusion formation, thus halting TEM at the apical EC membrane. Thus, post-arrest, cancer cells protrude through the EC cell-cell junctions and bind to basement membrane components via β_1_ integrin activation, push cell contents to the extravascular space, and further open the cell-cell junction to allow for nuclear transmigration (3, 79, 116).

During the last push across the endothelium, constrictive spaces (i.e., endothelial junctions) require the facilitation of critical mechanisms including active cancer cell nucleus deformation, cytoskeleton rearrangement and contraction, and nuclear softening. In these scenarios, cytoskeleton rearrangement, and contraction provides the mechanical forces for nuclear deformation, while softening of the nuclei due to the phosphorylation of Lamin A/C enables severe nuclear deformation (117–119). In some cases, severe nuclear deformation can lead to nuclear envelope rupture, which in turn can results in DNA damages due to uncontrolled exchange between the nucleus and the cytoplasm, as well as chromatin protrusion and fragmentation (120–122). Much more can be studied on the effects of nuclear confinement in metastasis, particularly how cancer cells overcome this barrier, using novel *in vitro* platforms.

Overall, TEM during metastasis constitutes a key step in the dissemination of carcinomas. To date, *in vitro* models have greatly influenced our understanding of cancer cell TEM. How the endothelium regulates this process directly remains to be studied. Developing cancer cell TEM platforms that rapidly detect instances of cell TEM may open the door to high-throughput platforms on which numerous drug candidates can be scanned for their ability to halt metastatic cancer cells at the apical EC surface during this critical step in the metastatic cascade.

## Emerging Topics in Transendothelial Migration

### Novel Cell Transendothelial Migration for Therapeutics

Developing novel molecular compounds for the inhibition or promotion of immune and cancer cell TEM has and will continue to be a high interest target in the drug discovery field. As our ability to isolate and manipulate cells for therapeutic applications advances, the avenues for regenerative medicine approaches may expand beyond tradition transplantation, and thus exist as an alternative to pharmaceutical treatment of tissue damage. These advances may permit the regeneration of damaged tissues or organs from primary donor or stem cells. One barrier to this progress may be our understanding of systemic administration of exogenous cells, specifically how we may manipulate these cells to quickly and non-disruptively pass the vascular endothelium to the site of tissue injury. To this end, microfluidic devices modeling novel donor or stem cell TEM may prove immensely beneficial in advancing our understanding of these potential processes.

Medicinal Signaling Cells (MSCs) (123) have been extensively studied in cell-based therapies for their immunomodulatory, immunosuppressive, and regenerative characteristics ideal for inflammatory, immune-mediated, and degenerative diseases. Various experimental therapies are underway for diseases of the musculoskeletal system (124), immune disorders (125), and even tumors (126). MSCs are derived from adult tissues (bone marrow, peripheral blood and adipose tissue) or from birth-associated tissues (placenta, amnion, umbilical cord and cord blood) which can differentiate into multiple lineages including osteocytes, adipocytes, and chondrocytes *in vitro. In vivo* MSCs do not function as tissue progenitors, but as secretory cells that induce the body's regenerative potential as a member of the universal stem cell niche present in tissues. A vital aspect of this regenerative effect is the endogenous and exogenous MSC ability to home into injured sites with high cytokine, chemokine and factor concentrations. This homing mechanism is defined as the active or passive arrest of the MSC within the vasculature followed by transmigration across the endothelium (127). Extensively described in its similarity to leukocyte transmigration, MSC homing is hypothesized to be comprised of the same five stages observed in leukocyte extravasation (circulation, rolling, adhesion, crawling, and TEM). Differences arise in the circulation stage where MSCs can experience impediments due to their larger size which can disrupt blood flow in small vessels and cause microembolisms (128–130). Recent observations show MSCs flattening to reduce the obstruction, yet the timing or mechanism of this passive arrest are not understood and can introduce serious consequences. MSCs differ in the crawling phase as well where they statically form cup-like protrusions into the endothelial barrier to aid in their TEM either transcellularly or through integration intracellularly. Enhancing TEM specificity of MSCs to the wound site is key to increasing the innate regenerative potential in each tissue (131). There are certain risks which still need to be overcome in MSC therapies including tumorigenicity (132), pro-inflammation and the increased risk of fibrosis with recurring treatments (133). Nonetheless, there are exciting clinical trials in the autologous and allogenic transplantations of functionalized MSCs for various diseases (ClinicalTrials.gov, key word MSC, Active).

While the plasticity and versatility of stems cells make them an obvious target of interest for tissue regeneration, primary, terminally differentiated cells also have the ability to aid in wound healing. Primary liver cells, hepatocytes, are a key example of primary cells with high regenerative capacity, studied extensively to date. Hepatocyte transplantation (HT) is a promising alternative approach to orthotopic liver transplantation for the treatment of acute liver failure (134–137) and inherited metabolic diseases of the liver (138–141). The aim of HT is the replacement of 5–15% of the native liver mass with healthy donor hepatocytes to restore physiological liver function (142, 143). As a result of three decades of research and development, HT is established as a safe procedure and performed clinically in over 100 cases internationally that have resulted in a bridge to liver transplantation or full recovery in many patients (144, 145). In contrast to orthotopic liver transplantation, HT is less invasive, enables multiple recipients to be transplanted from a single donor organ while the recipient's organ is not removed for faster recovery, cells can be cryopreserved and stored, and the procedure can be repeated if needed.

**Figure 3:**
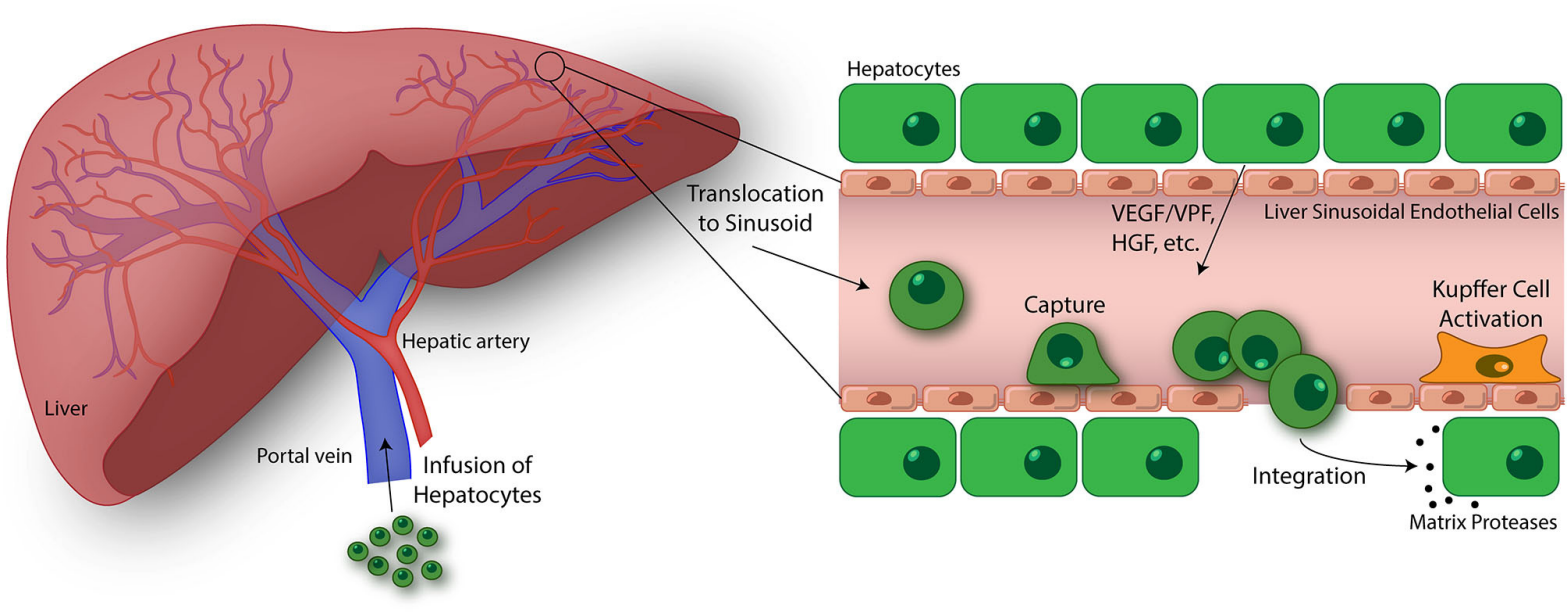
Emerging topics in the study of transendothelial migration (TEM). Hepatocyte transplantation is a promising treatment for liver disease and failure. In this process, donor hepatocytes are perfused into the portal vein where they may translocate to the liver sinusoids. Similar to cancer cells, size restrictions in the liver microvasculature, as well as potential integrin interactions, trap the hepatocytes at the apical endothelial cell surface. Entrapment resulting in ischemia-reperfusion events and Kupffer cell activation lead to liver sinusoidal endothelium disruption. Additionally, vascular endothelial growth factor (VEGF)/vascular permeability factor (VPF), hepatocyte growth factor (HGF), and other factors released by native hepatocytes in the liver plate further drive endothelium disruption, permitting passage of transfused hepatocytes. Once across, donor hepatocytes integrate with native hepatocytes as mediated through the activation of matrix proteases and separation of gap junctions (146). The ultimate goal of this process is to engraft these donor hepatocytes into the liver parenchyma and restore liver function. While there has been some success using this treatment strategy, an improved understanding of the hepatocyte TEM process may lead to discoveries promoting enhanced engraftment of hepatocytes in the liver. Using *in vitro* devices to breakdown and study this process is key to this progress.

The HT procedure involves isolating hepatocytes from donor liver deemed unsuitable for transplantation or surplus liver tissue from a healthy individual using collagenase perfusion, followed by injection into the portal vein (Figure 3) (147). Upon injection, transplanted hepatocytes migrate into liver sinusoids where they become entrapped, but must attach to the sinusoidal endothelium as well as undergo migration across the perisinusoidal space, separating the hepatocytes from the sinusoid, to engraft in the liver parenchyma (148, 149). The cells unable to quickly migrate across the endothelial barrier to integrate into the liver plates, are attacked and destroyed by immune cells. The immune response results in 70% loss of transplanted cells within 24–48 h after infusion (150, 151). Once in the liver parenchyma, the transplanted cells are stimulated by hormone and growth factors, and cell-cell and cell-extracellular matrix interactions critical for cell survival in the host (149). In addition, it is essential that engrafted cells are induced to proliferate following transplantation, which occurs under certain disease conditions that confers a selective growth advantage to donor hepatocytes (152), portal vein occlusion (153, 154), partial hepatectomy (155), and native liver irradiation (156). In mice, only 0.1–0.3% of donor hepatocytes engraft following infusion (157), highlighting the importance of engrafted hepatocytes undergoing expansion in the host liver. Prior studies conducted in rats have shown that sinusoidal endothelial disruption is a critical step for the entry of donor hepatocytes into the perisinusoidal space and subsequent engraftment using pharmacological agents and low-dose irradiation (136, 156, 158). Although augmenting engraftment, these approaches are toxic and not used clinically. Further, the attachment of hepatocytes to the sinusoidal endothelium modulates cell engraftment. The administration of fibronectin-like polymer prior to donor hepatocyte infusion was shown to enhance engraftment, suggesting that integrin-mediated interactions are essential for hepatocyte binding and migration through the sinusoidal endothelial barrier (159). However, the biophysical mechanisms of migration by hepatocytes and the time scales by which they occur have not been characterized.

Studies of hepatocyte migration across the sinusoidal endothelial barrier within an *in vitro* model would facilitate rapid screening of strategies for hepatocytes to evade immune responses and accelerate disruption of the sinusoidal endothelial layer. In addition, these *in vitro* models can potentially be used to facilitate screening of quality hepatocytes for clinical transplantation. A vast interest lies in gene editing using CRISPR-Cas technology in autologous hepatocytes for gene therapy targeting inherited metabolic diseases affecting the liver, which has been effective in animal models (160–162) and is the aim for future therapies. The advantage of the gene editing approach is it eliminates the need for immunosuppression and risk of graft rejection as cells isolated from the patient's resected liver are substrates for transplantation or hepatocytes may be edited *in vivo* to correct a monogenetic disease. In addition, gene editing has been used to introduce selection markers to make hepatocytes resistant to a small-molecule inhibitor for inducing gene modified hepatocytes to proliferate following engraftment in preclinical studies (163). As an alternative to fully differentiated hepatocytes, patient-derived reprogrammed iPSCs gene edited and differentiated into hepatocytes have the potential to be used for transplantation in the liver as demonstrated in preclinical studies in a mouse model of hemophilia B (164). Combining gene editing with *in vitro* assays would facilitate studies to identify genes that determine the efficiency of migration by hepatocytes across the sinusoidal endothelial barrier, such that precisely altering their expression using gene editing results in improved hepatocyte engraftment.

In the pursuit of novel *in vitro* platforms to study hepatocyte TEM, it is important to highlight EC heterogeneity [reviewed in depth previously (29)]. While a majority of leukocyte TEM occurs in the post-capillary venules (165), the liver (as well as the lung) is unique in that inflammation induces TEM at the capillary bed (166). In this region of the liver, sinusoidal ECs differ from traditional post-capillary venule ECs in that they are highly fenestrated, and have a comparatively discontinuous basement membrane, across from which hepatocytes line (167). This multi-cellular complex (like that of the brain and lung) permits communication between hepatocytes and the sinusoidal ECs. In the case of HT, it is important to take into account all of the differing factors when constructing an explorative platform. In a broader sense, while easy to obtain cell lines like HUVECs have traditionally been used for *in vitro* studies of TEM, EC heterogeneity must be considered when constructing *in vitro* models of the vasculature in key organ systems such as the liver, brain, and lung. Much like the pursuit of novel HT strategies, a better understanding of EC heterogeneity may permit the development of novel anti-inflammatories or migrastatics with effects targeting clinically high-risk organs (lung and brain). To conclude, HT is a prominent example of novel primary cell TEM that, if better understood, can have a profound impact in the clinic. Designing and building *in vitro* platforms that carefully represent the physiology of the vascular system of interest is key in bringing to light these promising therapeutics.

### Porous Membranes

**Figure 4:**
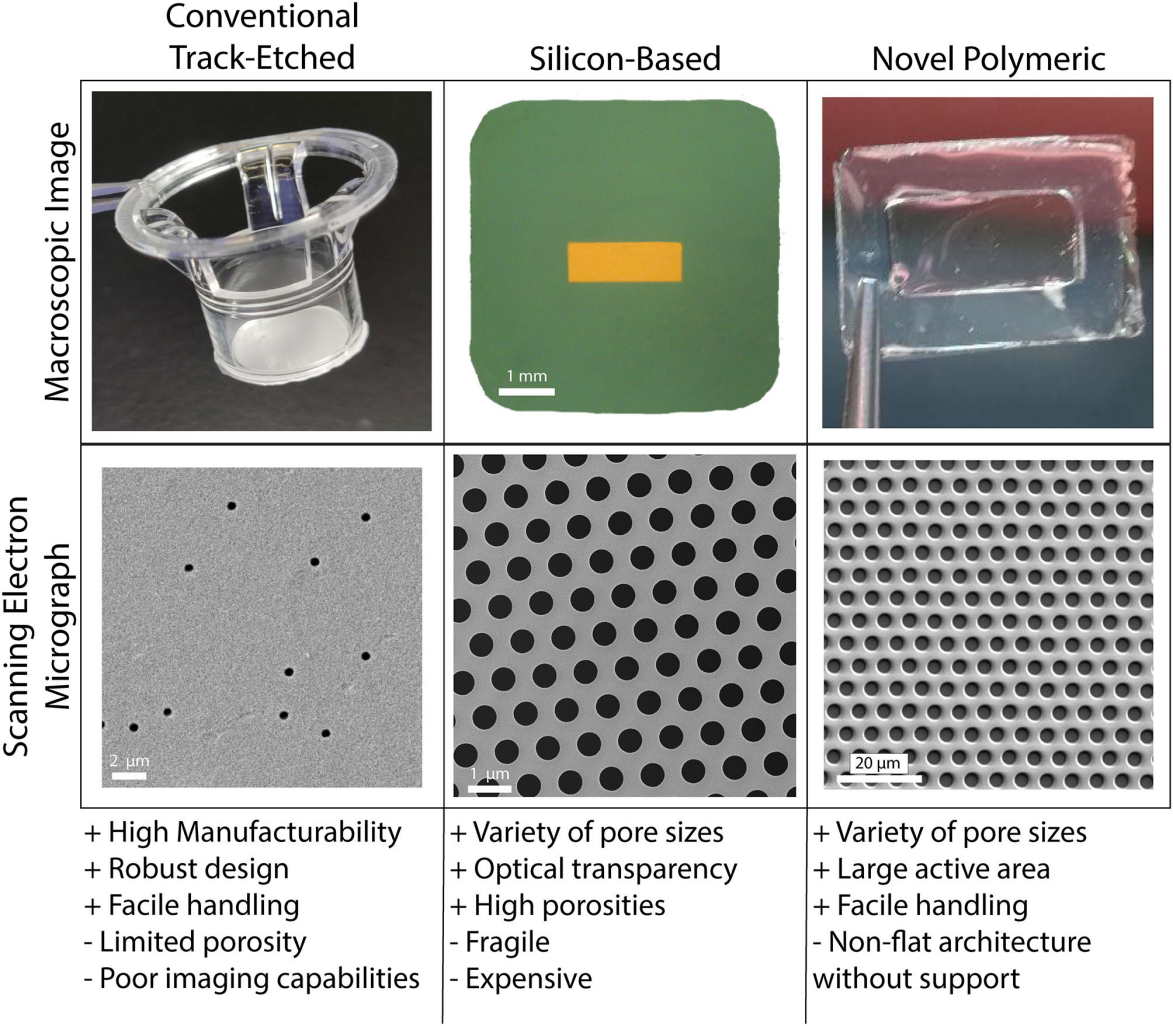
Porous cell culture support membranes are a vital and ever evolving feature of vascular mimetics for transmigration studies. The three main categories of porous membranes are conventional track-etched, silicon-based, and novel polymeric. Each of the main categories have pros (+) and cons (–) that may be weighed in the early stages of planning experimentation. Conventional track-etched membranes (polycarbonate filter shown here) have aided in years of transmigration studies, however, the onboarding of silicon-based (porous silicon nitride shown here) and novel polymeric (parylene membrane shown here) membranes has given way to many options for use within this field. Balancing usability and affordability will be key for drug discovery platforms.

As true for many of the works reviewed herein, porous membranes are vital components of *in vitro* human barrier models. These membranes enable multi-compartmental cell culture and they may be engineered to facilitate physiological cell-cell communication and cell transmigration (17, 168, 169). Establishing an *in vitro* barrier model that accurately recapitulates the physiological conditions of barrier systems *in vivo* requires high-porosity membranes with sub-micron thicknesses and precise pore geometries (170, 171). In general, these membranes should be mechanically robust, optically transparent, and they should sustain cell attachment and growth from relatively low cell density to confluency (169, 170, 172). Despite a historical abundance of studies on cell transmigration through human barrier models with porous membrane supports, membrane technologies continue to advance and diversify (Figure 4), facilitating elevated barrier model construction and diversifying applications. Thus, identifying the types of porous membranes available and the uses they offer may permit facile barrier model development in future studies of TEM.

Conventional polymeric membranes fabricated through a process called track-etching are the most widely used membranes for tissue-on-a-chip and human barrier models. These membranes can be made with a variety of pore sizes, ranging from submicron to 8 μm. Given the ease of handling and robust design, these commercially available membranes are suitable for a variety of tissue-chip applications. However, limitations in design and use motivate the exploration into novel membrane technologies for commercial use. Notably, due to the application of random irradiation to create pores, the track-etching process leads to membranes with very low porosity (often <10%) with random pore distributions. Attempts to increase track-etch membrane porosity leads to an increased chance of merged pores and even more variability (173, 174). Additionally, the relatively thick membrane structure is limited in physiological relevance. For instance, ECs and astrocytes (or glial cells) in the Blood-Brain Barrier (BBB) are separated by <300 nm, while the thickness of track-etched membranes used to recapitulate this tissue barrier often exceed 10 μm (14, 173). When considering experimental potential, another notable deficiency is the lack of optical transparency for imaging purposes which limits high-resolution imaging (173, 175). While imaging may not always be the focus of a respective study, the benefits of porous membranes with high-resolution imaging potential are immense in the context of transmigration studies.

Materials that have been proposed to substitute conventional track-etched membranes include novel polymeric thin films such as polydimethylsiloxane (PDMS) elastomer (176) and silicon-based thin layers such as silicon nitride (15). Among silicon-based materials, porous nanocrystalline silicon (pnc-Si), Silicon Oxide (SiO2), and Silicon Nitride (Si3N4) are promising candidates which were successfully used by our group to fabricate membranes for *in vitro* modeling of tissue-on-a-chip and barrier systems. These membranes provided physiologically relevant thickness in the submicron region, optical transparency, and controlled pore size and pore distribution (17, 175, 177, 178). Notable examples of novel polymeric membranes for *in vitro* studies include PDMS, parylene, and poly(carbonate) (PC). For example, one of the pioneering studies for reconstructing the alveolar-blood interface of the lung involved casting PDMS on 10 μm pillars to fabricate porous membranes (179). Like conventional polymeric track-etch membranes, however, both these classes of materials possess advantages and disadvantages that should be considered and potentially addressed for specific applications. In general, novel polymeric membranes tend to adopt non-flat shapes, which makes cell culture and microscopy challenging (174). They also have limited resistance to changes in temperature and pressure (180). Silicon-based membranes are more chemically and thermally stable and have superior permeability, but are considered fragile and rigid (180, 181). Furthermore, expensive processing of silicon limits membrane manufacturability. Despite these material pitfalls, there are extra (and often straightforward) steps such as chemical treatment, physical reinforcement, introducing a support layer and modifications in the fabrication strategies that can mitigate and potentially eliminate these disadvantages. Much like the construction of a microphysiological system itself, finding the balance between usability and complexity/physiological relevance when fabricating porous membranes for transmigration studies is key to progressing this field and opening the door to novel drug discovery platforms.

## Conclusion

*In vitro* platforms have and continue to guide biological discovery in a variety of fields. Given the complexity and imaging needs to efficiently study cell TEM, *in vitro* platforms provide labs with the unique opportunity to better our understanding of this process across a variety of migratory cell types. In many cases, the process of TEM can be exploited as a limiting step in disease pathogenesis. Insufficient or maladaptive leukocyte transmigration drives inflammatory disease and immunodeficient patient outcomes. Additionally, cancer cell TEM, both in intravasation and extravasation, constitutes a major step in metastasis. Lastly, systemic delivery of MSCs or donor hepatocytes, for example, signifies the ability to exploit TEM for regenerative medicine applications. Together, our understanding of any of these TEM processes would not be the level it is without the use of traditional and advanced *in vitro* platforms. With drug discovery costs rising and applicant compounds decreasing, the potential to cost-effectively and accurately screen drug candidates using these *in vitro* platforms may be the spark needed to drive pharmaceutical research and development forward.

## Author Contributions

AS conceptualized, wrote, and edited the manuscript. ZA, SG, MM, and RA contributed writing to the manuscript. RC conceptualized sections and contributed writing to the manuscript. TG and JM conceptualized and significantly edited the manuscript. All authors reviewed the final manuscript.

## Conflict of Interest

TG and JM are co-founders of SiMPore, an early-stage company commercializing ultrathin silicon-based technologies. The remaining authors declare that the research was conducted in the absence of any commercial or financial relationships that could be construed as a potential conflict of interest.

## References

[1] Trounson A Thakar RG Lomax G Gibbons D. Clinical trials for stem cell therapies. BMC Med. (2011) 9:52. 10.1186/1741-7015-9-52 21569277 PMC3098796

[2] Ley K Laudanna C Cybulsky MI Nourshargh S. Getting to the site of inflammation: the leukocyte adhesion cascade updated. Nat Rev Immunol. (2007) 7:678–89. 10.1038/nri2156 17717539

[3] Chen MB Whisler JA Jeon JS Kamm RD. Mechanisms of tumor cell extravasation in an *in vitro* microvascular network platform. Integr Biol. (2013) 5:1262–71. 10.1039/c3ib40149a 23995847 PMC4038741

[4] Hay M Thomas DW Craighead JL Economides C Rosenthal J. Clinical development success rates for investigational drugs. Nat Biotechnol. (2014) 32:40–51. 10.1038/nbt.2786 24406927

[5] Seok J Warren HS Cuenca AG Mindrinos MN Baker HV Xu W. Genomic responses in mouse models poorly mimic human inflammatory diseases. Proc Natl Acad Sci USA. (2013) 110:3507. 10.1073/pnas.1222878110 23401516 PMC3587220

[6] Ranieri VM Thompson BT Barie PS Dhainaut JF Douglas IS Finfer S. Drotrecogin Alfa. (activated) in adults with septic shock. N Engl J Med. (2012) 366:2055–64. 10.1056/NEJMoa1202290 22616830

[7] Mackay CR. Moving targets: cell migration inhibitors as new anti-inflammatory therapies. Nat Immunol. (2008) 9:988–98. 10.1038/ni.f.210 18711436

[8] Lerman YV Kim M. Neutrophil migration under normal and sepsis conditions. Cardiovasc Hematol Disord Drug Targets. (2015) 15:19–28. 10.2174/1871529X15666150108113236 25567338 PMC5111082

[9] Allingham MJ van Buul JD Burridge K. ICAM-1-mediated, Src- and Pyk2-dependent vascular endothelial cadherin tyrosine phosphorylation is required for leukocyte transendothelial migration. J Immunol. (2007) 179:4053. 10.4049/jimmunol.179.6.4053 17785844

[10] Bixel MG Li H Petri B Khandoga AG Khandoga A Zarbock A. CD99 and CD99L2 act at the same site as, but independently of, PECAM-1 during leukocyte diapedesis. Blood. (2010) 116:1172–84. 10.1182/blood-2009-12-256388 20479283

[11] Buffone A Anderson NR Hammer DA. Human neutrophils will crawl upstream on ICAM-1 if Mac-1 is blocked. Biophys J. (2019) 117:1393–404. 10.1016/j.bpj.2019.08.044 31585707 PMC6817642

[12] Carman CV Sage PT Sciuto TE de la Fuente MA Geha RS Ochs HD. Transcellular diapedesis is initiated by invasive podosomes. Immunity. (2007) 26:784–97. 10.1016/j.immuni.2007.04.015 17570692 PMC2094044

[13] Green CE Pearson DN Camphausen RT Staunton DE Simon SI. Shear-dependent capping of L-selectin and P-selectin glycoprotein ligand 1 by E-selectin signals activation of high-avidity beta2-integrin on neutrophils. J Immunol. (2004) 172:7780–90. 10.4049/jimmunol.172.12.7780 15187162

[14] Khire TS Salminen AT Swamy H Lucas KS McCloskey MC Ajalik RE. Microvascular mimetics for the study of leukocyte–endothelial interactions. Cell Mol Bioeng. (2020) 13:125–39. 10.1007/s12195-020-00611-6 32175026 PMC7048879

[15] Mossu A Rosito M Khire T Li Chung H Nishihara H Gruber I. A silicon nanomembrane platform for the visualization of immune cell trafficking across the human blood–brain barrier under flow. J Cereb Blood Flow Metab. (2019) 39:395–410. 10.1177/0271678X18820584 30565961 PMC6421249

[16] O'Brien CD Lim P Sun J Albelda SM. PECAM-1-dependent neutrophil transmigration is independent of monolayer PECAM-1 signaling or localization. Blood. (2003) 101:2816–25. 10.1182/blood-2002-08-2396 12468430

[17] Salminen AT Zhang J Madejski GR Khire TS Waugh RE McGrath JL. Ultrathin dual-scale nano- and microporous membranes for vascular transmigration models. Small. (2019) 15:e1804111. 10.1002/smll.201804111 30632319 PMC6530565

[18] Shaw SK Bamba PS Perkins BN Luscinskas FW. Real-time imaging of vascular endothelial-cadherin during leukocyte transmigration across endothelium. J Immunol. (2001) 167:2323–30. 10.4049/jimmunol.167.4.2323 11490021

[19] Steiner O Coisne C Cecchelli R Boscacci R Deutsch U Engelhardt B. Differential roles for endothelial ICAM-1, ICAM-2, and VCAM-1 in shear-resistant T cell arrest, polarization, and directed crawling on blood–brain barrier endothelium. J Immunol. (2010) 185:4846–55. 10.4049/jimmunol.0903732 20861356

[20] Watson RL Buck J Levin LR Winger RC Wang J Arase H. Endothelial CD99 signals through soluble adenylyl cyclase and PKA to regulate leukocyte transendothelial migration. J Exp Med. (2015) 212:1021–41. 10.1084/jem.20150354 26101266 PMC4493416

[21] Yang L Froio RM Sciuto TE Dvorak AM Alon R Luscinskas FW. ICAM-1 regulates neutrophil adhesion and transcellular migration of TNF-alpha-activated vascular endothelium under flow. Blood. (2005) 106:584–92. 10.1182/blood-2004-12-4942 15811956 PMC1635241

[22] Jones DA McIntire LV Smith CW Picker LJ. A two-step adhesion cascade for T cell/endothelial cell interactions under flow conditions. J Clin Invest. (1994) 94:2443–50. 10.1172/JCI117612 7527432 PMC330076

[23] Luscinskas FW Ding H Lichtman AH. P-selectin and vascular cell adhesion molecule 1 mediate rolling and arrest, respectively, of CD4+ T lymphocytes on tumor necrosis factor alpha-activated vascular endothelium under flow. J Exp Med. (1995) 181:1179–86. 10.1084/jem.181.3.1179 7532680 PMC2191919

[24] Bahra P Rainger GE Wautier JL Nguyet-Thin L Nash GB. Each step during transendothelial migration of flowing neutrophils is regulated by the stimulatory concentration of tumour necrosis factor-alpha. Cell Adhes Commun. (1998) 6:491–501. 10.3109/15419069809010797 9929742

[25] Nourshargh S Hordijk PL Sixt M. Breaching multiple barriers: leukocyte motility through venular walls and the interstitium. Nat Rev Mol Cell Biol. (2010) 11:366–78. 10.1038/nrm2889 20414258

[26] Schmidt EP Yang Y Janssen WJ Gandjeva A Perez MJ Barthel L. The pulmonary endothelial glycocalyx regulates neutrophil adhesion and lung injury during experimental sepsis. Nat Med. (2012) 18:1217–23. 10.1038/nm.2843 22820644 PMC3723751

[27] McDonald KK Cooper S Danielzak L Leask RL. Glycocalyx degradation induces a proinflammatory phenotype and increased leukocyte adhesion in cultured endothelial cells under flow. PLoS ONE. (2016) 11:e0167576. 10.1371/journal.pone.0167576 27907146 PMC5132265

[28] Jones DA Smith CW McIntire LV. Leucocyte adhesion under flow conditions: principles important in tissue engineering. Biomaterials. (1996) 17:337–47. 10.1016/0142-9612(96)85572-4 8745331

[29] Aird WC. Phenotypic heterogeneity of the endothelium: I. structure, function, and mechanisms. Circ Res. (2007) 100:158–73. 10.1161/01.RES.0000255691.76142.4a 17272818

[30] Nourshargh S Alon R. Leukocyte migration into inflamed tissues. Immunity. (2014) 41:694–707. 10.1016/j.immuni.2014.10.008 25517612

[31] Laubli H Borsig L. Selectins promote tumor metastasis. Semin Cancer Biol. (2010) 20:169–77. 10.1016/j.semcancer.2010.04.005 20452433

[32] Zarbock A Ley K McEver RP Hidalgo A. Leukocyte ligands for endothelial selectins: specialized glycoconjugates that mediate rolling and signaling under flow. Blood. (2011) 118:6743–51. 10.1182/blood-2011-07-343566 22021370 PMC3245201

[33] Anderson NR Lee D Hammer DA. An experimentally determined state diagram for human CD4(+) T lymphocyte CXCR4-stimulated adhesion under shear flow. Cell Mol Bioeng. (2018) 11:91–8. 10.1007/s12195-018-0519-x 30271505 PMC6157745

[34] Sheikh S Rainger GE Gale Z Rahman M Nash GB. Exposure to fluid shear stress modulates the ability of endothelial cells to recruit neutrophils in response to tumor necrosis factor-alpha: a basis for local variations in vascular sensitivity to inflammation. Blood. (2003) 102:2828–34. 10.1182/blood-2003-01-0080 12829609

[35] Yang H Li N Du Y Tong C Lu S Hu J. Neutrophil adhesion and crawling dynamics on liver sinusoidal endothelial cells under shear flow. Exp Cell Res. (2017) 351:91–9. 10.1016/j.yexcr.2017.01.002 28077302

[36] Aird WC. Spatial and temporal dynamics of the endothelium. J Thromb Haemost. (2005) 3:1392–406. 10.1111/j.1538-7836.2005.01328.x 15892866

[37] Benson BL Li L Myers JT Dorand RD Gurkan UA Huang AY. Biomimetic post-capillary venule expansions for leukocyte adhesion studies. Sci Rep. (2018) 8:9328. 10.1038/s41598-018-27566-z 29921896 PMC6008471

[38] Prabhakarpandian B Pant K Scott RC Pattillo CB Irimia D Kiani MF. Synthetic microvascular networks for quantitative analysis of particle adhesion. Biomed Microdevices. (2008) 10:585–95. 10.1007/s10544-008-9170-y 18327641 PMC4476637

[39] Santaguida S Janigro D Hossain M Oby E Rapp E Cucullo L. Side by side comparison between dynamic versus static models of blood-brain barrier *in vitro*: a permeability study. Brain Res. (2006) 1109:1–13. 10.1016/j.brainres.2006.06.027 16857178

[40] Voyvodic PL Min D Baker AB. A multichannel dampened flow system for studies on shear stress-mediated mechanotransduction. Lab Chip. (2012) 12:3322–30. 10.1039/c2lc40526a 22836694 PMC3426609

[41] Wevers NR Kasi DG Gray T Wilschut KJ Smith B van Vught R. A perfused human blood-brain barrier on-a-chip for high-throughput assessment of barrier function and antibody transport. Fluids Barriers CNS. (2018) 15:23. 10.1186/s12987-018-0108-3 30165870 PMC6117964

[42] Kunkel EJ Dunne JL Ley K. Leukocyte arrest during cytokine-dependent inflammation *in vivo*. J Immunol. (2000) 164:3301–8. 10.4049/jimmunol.164.6.3301 10706723

[43] Montresor A Toffali L Constantin G Laudanna C. Chemokines and the signaling modules regulating integrin affinity. Front Immunol. (2012) 3:127. 10.3389/fimmu.2012.00127 22654882 PMC3360201

[44] Faull RJ Ginsberg MH. Inside-out signaling through integrins. J Am Soc Nephrol. (1996) 7:1091–7. 8866399 10.1681/ASN.V781091

[45] Fagerholm SC Guenther C Llort Asens M Savinko T Uotila LM. Beta2-integrins and interacting proteins in leukocyte trafficking, immune suppression, and immunodeficiency disease. Front Immunol. (2019) 10:254. 10.3389/fimmu.2019.00254 30837997 PMC6389632

[46] Thelen M Stein JV. How chemokines invite leukocytes to dance. Nat Immunol. (2008) 9:953–9. 10.1038/ni.f.207 18711432

[47] Huber AR Kunkel SL Todd RF III Weiss SJ. Regulation of transendothelial neutrophil migration by endogenous interleukin-8. Science. (1991) 254:99–102. 10.1126/science.1718038 1718038

[48] Luscinskas FW Kiely JM Ding H Obin MS Hebert CA Baker JB. *In vitro* inhibitory effect of IL-8 and other chemoattractants on neutrophil-endothelial adhesive interactions. J Immunol. (1992) 149:2163–71. 1381398

[49] Smart SJ Casale TB. TNF-alpha-induced transendothelial neutrophil migration is IL-8 dependent. Am J Physiol. (1994) 266 (3 Pt. 1):L238–45. 10.1152/ajplung.1994.266.3.L238 8166294

[50] Hol J Wilhelmsen L Haraldsen G. The murine IL-8 homologues KC, MIP-2, and LIX are found in endothelial cytoplasmic granules but not in Weibel-Palade bodies. J Leukoc Biol. (2010) 87:501–8. 10.1189/jlb.0809532 20007247

[51] DiVietro JA Smith MJ Smith BRE Petruzzelli L Larson RS Lawrence MB. Immobilized IL-8 triggers progressive activation of neutrophils rolling *in vitro* on P-selectin and intercellular adhesion molecule-1. J Immunol. (2001) 167:4017. 10.4049/jimmunol.167.7.4017 11564821

[52] Pichert A Samsonov SA Theisgen S Thomas L Baumann L Schiller J. Characterization of the interaction of interleukin-8 with hyaluronan, chondroitin sulfate, dermatan sulfate and their sulfated derivatives by spectroscopy and molecular modeling. Glycobiology. (2012) 22:134–45. 10.1093/glycob/cwr120 21873605 PMC3230280

[53] Gerszten RE Garcia-Zepeda EA Lim YC Yoshida M Ding HA Gimbrone MA. MCP-1 and IL-8 trigger firm adhesion of monocytes to vascular endothelium under flow conditions. Nature. (1999) 398:718–23. 10.1038/19546 10227295

[54] Ulfman LH Joosten DPH van der Linden JAM Lammers JWJ Zwaginga JJ Koenderman L. IL-8 induces a transient arrest of rolling eosinophils on human endothelial cells. J Immunol. (2001) 166:588. 10.4049/jimmunol.166.1.588 11123341

[55] Moser B Willimann K. Chemokines: role in inflammation and immune surveillance. Ann Rheum Dis. (2004) 63 (Suppl. 2):ii84–9. 10.1136/ard.2004.028316 15479880 PMC1766778

[56] Kim D Haynes CL. Neutrophil chemotaxis within a competing gradient of chemoattractants. Anal Chem. (2012) 84:6070–8. 10.1021/ac3009548 22816782 PMC3404751

[57] Collins SJ Ruscetti FW Gallagher RE Gallo RC. Normal functional characteristics of cultured human promyelocytic leukemia cells. (HL-60) after induction of differentiation by dimethylsulfoxide. J Exp Med. (1979) 149:969–74. 10.1084/jem.149.4.969 219131 PMC2184853

[58] Martin SJ Bradley JG Cotter TG. HL-60 cells induced to differentiate towards neutrophils subsequently die via apoptosis. Clin Exp Immunol. (1990) 79:448–53. 10.1111/j.1365-2249.1990.tb08110.x 2317949 PMC1534969

[59] Bartholomäus I Kawakami N Odoardi F Schläger C Miljkovic D Ellwart JW. Effector T cell interactions with meningeal vascular structures in nascent autoimmune CNS lesions. Nature. (2009) 462:94–8. 10.1038/nature08478 19829296

[60] Issekutz AC Rowter D Springer TA. Role of ICAM-1 and ICAM-2 and alternate CD11/CD18 ligands in neutrophil transendothelial migration. J Leukoc Biol. (1999) 65:117–26. 10.1002/jlb.65.1.117 9886254

[61] Lyck R Enzmann G. The physiological roles of ICAM-1 and ICAM-2 in neutrophil migration into tissues. Curr Opin Hematol. (2015) 22:53–9. 10.1097/MOH.0000000000000103 25427141

[62] Li N Yang H Wang M Lü S Zhang Y Long M. Ligand-specific binding forces of LFA-1 and Mac-1 in neutrophil adhesion and crawling. Mol Biol Cell. (2017) 29:408–18. 10.1091/mbc.E16-12-0827 29282280 PMC6014170

[63] Carman CV Springer TA. A transmigratory cup in leukocyte diapedesis both through individual vascular endothelial cells and between them. J Cell Biol. (2004) 167:377–88. 10.1083/jcb.200404129 15504916 PMC2172560

[64] Privratsky JR Newman DK Newman PJ. PECAM-1: conflicts of interest in inflammation. Life Sci. (2010) 87:69–82. 10.1016/j.lfs.2010.06.001 20541560 PMC2917326

[65] Newman Peter J Newman Debra K. Signal transduction pathways mediated by PECAM-1. Arterioscler Thromb Vasc Biol. (2003) 23:953–64. 10.1161/01.ATV.0000071347.69358.D9 12689916

[66] Muller WA Weigl SA Deng X Phillips DM. PECAM-1 is required for transendothelial migration of leukocytes. J Exp Med. (1993) 178:449–60. 10.1084/jem.178.2.449 8340753 PMC2191108

[67] Gonzalez AM Cyrus BF Muller WA. Targeted recycling of the lateral border recycling compartment precedes adherens junction dissociation during transendothelial migration. Am J Pathol. (2016) 186:1387–402. 10.1016/j.ajpath.2016.01.010 26968345 PMC4861767

[68] Mamdouh Z Chen X Pierini LM Maxfield FR Muller WA. Targeted recycling of PECAM from endothelial surface-connected compartments during diapedesis. Nature. (2003) 421:748–53. 10.1038/nature01300 12610627

[69] Mamdouh Z Mikhailov A Muller WA. Transcellular migration of leukocytes is mediated by the endothelial lateral border recycling compartment. J Exp Med. (2009) 206:2795. 10.1084/jem.20082745 19887395 PMC2806621

[70] Lou O Alcaide P Luscinskas FW Muller WA. CD99 is a key mediator of the transendothelial migration of neutrophils. J Immunol. (2007) 178:1136. 10.4049/jimmunol.178.2.1136 17202377

[71] Gavard J. Endothelial permeability and VE-cadherin: a wacky comradeship. Cell Adh Migr. (2014) 8:158–64. 10.4161/cam.29026 25422846 PMC4049861

[72] Muller WA. Localized signals that regulate transendothelial migration. Curr Opin Immunol. (2016) 38:24–9. 10.1016/j.coi.2015.10.006 26584476 PMC4715928

[73] Potter MD Barbero S Cheresh DA. Tyrosine phosphorylation of VE-cadherin prevents binding of p120- and beta-catenin and maintains the cellular mesenchymal state. J Biol Chem. (2005) 280:31906–12. 10.1074/jbc.M505568200 16027153

[74] Hamilla SM Stroka KM Aranda-Espinoza H. VE-cadherin-independent cancer cell incorporation into the vascular endothelium precedes transmigration. PLoS ONE. (2014) 9:e109748. 10.1371/journal.pone.0109748 25275457 PMC4183660

[75] Gandalovičová A Rosel D Fernandes M Veselý P Heneberg P Cermák V. Migrastatics-anti-metastatic and anti-invasion drugs: promises and challenges. Trends Cancer. (2017) 3:391–406. 10.1016/j.trecan.2017.04.008 28670628 PMC5482322

[76] Chen MB Lamar JM Li R Hynes RO Kamm RD. Elucidation of the roles of tumor integrin β1 in the extravasation stage of the metastasis cascade. Cancer Res. (2016) 76:2513–24. 10.1158/0008-5472.CAN-15-1325 26988988 PMC4873393

[77] Cui X Guo W Sun Y Sun B Hu S Sun D. A microfluidic device for isolation and characterization of transendothelial migrating cancer cells. Biomicrofluidics. (2017) 11:014105. 10.1063/1.4974012 28798840 PMC5533502

[78] Herman H Fazakas C Haskó J Molnár K Mészáros Á Nyúl-Tóth Á. Paracellular and transcellular migration of metastatic cells through the cerebral endothelium. J Cell Mol Med. (2019) 23:2619–31. 10.1111/jcmm.14156 30712288 PMC6433661

[79] Jeon JS Zervantonakis IK Chung S Kamm RD Charest JL. *In vitro* model of tumor cell extravasation. PLoS ONE. (2013) 8:e56910. 10.1371/journal.pone.0056910 23437268 PMC3577697

[80] Li YH Zhu C. A modified Boyden chamber assay for tumor cell transendothelial migration *in vitro*. Clin Exp Metastasis. (1999) 17:423–9. 10.1023/A:1006614232388 10651309

[81] Ni B-S Tzao C Huang J-H. Plug-and-play *in vitro* metastasis system toward recapitulating the metastatic cascade. Sci Rep. (2019) 9:18110. 10.1038/s41598-019-54711-z 31792319 PMC6889311

[82] Song JW Cavnar SP Walker AC Luker KE Gupta M Tung YC. Microfluidic endothelium for studying the intravascular adhesion of metastatic breast cancer cells. PLoS ONE. (2009) 4:e5756. 10.1371/journal.pone.0005756 19484126 PMC2684591

[83] Zervantonakis IK Hughes-Alford SK Charest JL Condeelis JS Gertler FB Kamm RD. Three-dimensional microfluidic model for tumor cell intravasation and endothelial barrier function. Proc Natl Acad Sci USA. (2012) 109:13515. 10.1073/pnas.1210182109 22869695 PMC3427099

[84] Dongre A Weinberg RA. New insights into the mechanisms of epithelial-mesenchymal transition and implications for cancer. Nat Rev Mol Cell Biol. (2019) 20:69–84. 10.1038/s41580-018-0080-4 30459476

[85] Ye X Tam WL Shibue T Kaygusuz Y Reinhardt F Ng Eaton E. Distinct EMT programs control normal mammary stem cells and tumour-initiating cells. Nature. (2015) 525:256–60. 10.1038/nature14897 26331542 PMC4764075

[86] Chaffer CL Weinberg RA. A perspective on cancer cell metastasis. Science. (2011) 331:1559. 10.1126/science.1203543 21436443

[87] Singh A Settleman J. EMT, cancer stem cells and drug resistance: an emerging axis of evil in the war on cancer. Oncogene. (2010) 29:4741–51. 10.1038/onc.2010.215 20531305 PMC3176718

[88] Krebs MG Hou J-M Ward TH Blackhall FH Dive C. Circulating tumour cells: their utility in cancer management and predicting outcomes. Ther Adv Med Oncol. (2010) 2:351–65. 10.1177/1758834010378414 21789147 PMC3126032

[89] Stemmler MP Eccles RL Brabletz S Brabletz T. Non-redundant functions of EMT transcription factors. Nat Cell Biol. (2019) 21:102–12. 10.1038/s41556-018-0196-y 30602760

[90] Choi B-J Park S-A Lee S-Y Cha YN Surh Y-J. Hypoxia induces epithelial-mesenchymal transition in colorectal cancer cells through ubiquitin-specific protease 47-mediated stabilization of Snail: a potential role of Sox9. Sci Rep. (2017) 7:15918. 10.1038/s41598-017-15139-5 29162839 PMC5698333

[91] Xu J Lamouille S Derynck R. TGF-beta-induced epithelial to mesenchymal transition. Cell Res. (2009) 19:156–72. 10.1038/cr.2009.5 19153598 PMC4720263

[92] Saitoh M. Involvement of partial EMT in cancer progression. J Biochem. (2018) 164:257–64. 10.1093/jb/mvy047 29726955

[93] Pal M Chen H Lee BH Lee JYH Yip YS Tan NS. Epithelial-mesenchymal transition of cancer cells using bioengineered hybrid scaffold composed of hydrogel/3D-fibrous framework. Sci Rep. (2019) 9:8997. 10.1038/s41598-019-45384-9 31222037 PMC6586872

[94] Ki CS Lin TY Korc M Lin CC. Thiol-ene hydrogels as desmoplasia-mimetic matrices for modeling pancreatic cancer cell growth, invasion, and drug resistance. Biomaterials. (2014) 35:9668–77. 10.1016/j.biomaterials.2014.08.014 25176061 PMC4164606

[95] Pisano M Triacca V Barbee KA Swartz MA. An *in vitro* model of the tumor–lymphatic microenvironment with simultaneous transendothelial and luminal flows reveals mechanisms of flow enhanced invasion. Integr Biol. (2015) 7:525–33. 10.1039/C5IB00085H 25896438

[96] Xu H Li Z Yu Y Sizdahkhani S Ho WS Yin F. A dynamic *in vivo*-like organotypic blood-brain barrier model to probe metastatic brain tumors. Sci Rep. (2016) 6:36670. 10.1038/srep36670 27830712 PMC5103210

[97] Xu Z Li E Guo Z Yu R Hao H Xu Y. Design and construction of a multi-organ microfluidic chip mimicking the *in vivo* microenvironment of lung cancer metastasis. ACS Appl Mater Interfaces. (2016) 8:25840–7. 10.1021/acsami.6b08746 27606718

[98] Anderson RL Balasas T Callaghan J Coombes RC Evans J Hall JA. A framework for the development of effective anti-metastatic agents. Nat Rev Clin Oncol. (2019) 16:185–204. 10.1038/s41571-018-0134-8 30514977 PMC7136167

[99] Strell C Entschladen F. Extravasation of leukocytes in comparison to tumor cells. Cell Commun Signal. (2008) 6:10. 10.1186/1478-811X-6-10 19055814 PMC2627905

[100] Shirure VS Reynolds NM Burdick MM. Mac-2 binding protein is a novel E-selectin ligand expressed by breast cancer cells. PLoS ONE. (2012) 7:e44529. 10.1371/journal.pone.0044529 22970241 PMC3435295

[101] Shea DJ Li YW Stebe KJ Konstantopoulos K. E-selectin-mediated rolling facilitates pancreatic cancer cell adhesion to hyaluronic acid. FASEB J. (2017) 31:5078–86. 10.1096/fj.201700331R 28765175 PMC5636711

[102] Reynolds NM Mohammadalipour A Hall CR Asghari Adib A Farnoud AM Burdick MM. Galectin-1 influences breast cancer cell adhesion to e-selectin via ligand intermediaries. Cell Mol Bioeng. (2018) 11:37–52. 10.1007/s12195-017-0512-9 31719877 PMC6816655

[103] Edwards EE Birmingham KG O'Melia MJ Oh J Thomas SN. Fluorometric quantification of single-cell velocities to investigate cancer metastasis. Cell Syst. (2018) 7:496–509.e6. 10.1016/j.cels.2018.10.005 30414924 PMC7254938

[104] Spencer A Baker AB. High throughput label free measurement of cancer cell adhesion kinetics under hemodynamic flow. Sci Rep. (2016) 6:19854. 10.1038/srep19854 26816215 PMC4728493

[105] Stoletov K Kato H Zardouzian E Kelber J Yang J Shattil S. Visualizing extravasation dynamics of metastatic tumor cells. J Cell Sci. (2010) 123 (Pt. 13):2332–41. 10.1242/jcs.069443 20530574 PMC2886748

[106] Price EA Coombe DR Murray JC. Beta-1 integrins mediate tumour cell adhesion to quiescent endothelial cells *in vitro*. Br J Cancer. (1996) 74:1762–6. 10.1038/bjc.1996.627 8956790 PMC2077205

[107] Hynes RO. Integrins: bidirectional, allosteric signaling machines. Cell. (2002) 110:673–87. 10.1016/S0092-8674(02)00971-6 12297042

[108] Weiss L Grundmann E Torhorst J Hartveit F Moberg I Eder M. Haematogenous metastatic patterns in colonic carcinoma: an analysis of 1541 necropsies. J Pathol. (1986) 150:195–203. 10.1002/path.1711500308 3806280

[109] Yamauchi K Yang M Jiang P Xu M Yamamoto N Tsuchiya H. Development of real-time subcellular dynamic multicolor imaging of cancer-cell trafficking in live mice with a variable-magnification whole-mouse imaging system. Cancer Res. (2006) 66:4208–14. 10.1158/0008-5472.CAN-05-3927 16618743

[110] Leong HS Robertson AE Stoletov K Leith SJ Chin CA Chien AE. (2014). Invadopodia are required for cancer cell extravasation and are a therapeutic target for metastasis. Cell Rep. (2014) 8:1558–70. 10.1016/j.celrep.2014.07.050 25176655

[111] Shin MK Kim SK Jung H. Integration of intra- and extravasation in one cell-based microfluidic chip for the study of cancer metastasis. Lab Chip. (2011) 11:3880–7. 10.1039/c1lc20671k 21975823

[112] Bapu D Runions J Kadhim M Brooks SA. N-acetylgalactosamine glycans function in cancer cell adhesion to endothelial cells: a role for truncated O-glycans in metastatic mechanisms. Cancer Lett. (2016) 375:367–74. 10.1016/j.canlet.2016.03.019 26994652

[113] Voura EB Sandig M Siu CH. Cell–cell interactions during transendothelial migration of tumor cells. Microsc Res Tech. (1998) 43:265–75. 10.1002/(SICI)1097-0029(19981101)43:3<265::AID-JEMT9>3.0.CO;2-Z 9840805

[114] Reymond N Im JH Garg R Vega FM Borda d'Agua B Riou P. Cdc42 promotes transendothelial migration of cancer cells through β1 integrin. J Cell Biol. (2012) 199:653–68. 10.1083/jcb.201205169 23148235 PMC3494849

[115] Fu Y Chin LK Bourouina T Liu AQ VanDongen AMJ. Nuclear deformation during breast cancer cell transmigration. Lab Chip. (2012) 12:3774–8. 10.1039/c2lc40477j 22864314

[116] Chen MB Whisler JA Fröse J Yu C Shin Y Kamm RD. On-chip human microvasculature assay for visualization and quantification of tumor cell extravasation dynamics. Nat Protoc. (2017) 12:865–80. 10.1038/nprot.2017.018 28358393 PMC5509465

[117] Dahl KN Ribeiro AJS Lammerding J. Nuclear shape, mechanics, and mechanotransduction. Circ Res. (2008) 102:1307–18. 10.1161/CIRCRESAHA.108.173989 18535268 PMC2717705

[118] Davidson PM Sliz J Isermann P Denais C Lammerding J. Design of a microfluidic device to quantify dynamic intra-nuclear deformation during cell migration through confining environments. Integrat Biol. (2015) 7:1534–46. 10.1039/C5IB00200A 26549481 PMC4666765

[119] Das A Barai A Monteiro M Kumar S Sen S. Nuclear softening is essential for protease-independent migration. Matrix Biol. (2019) 82:4–19. 10.1016/j.matbio.2019.01.001 30641137

[120] Denais CM Gilbert RM Isermann P McGregor AL Te Lindert M Weigelin B. Nuclear envelope rupture and repair during cancer cell migration. Science. (2016) 352:353–8. 10.1126/science.aad7297 27013428 PMC4833568

[121] Lammerding J Wolf K. Nuclear envelope rupture: actin fibers are putting the squeeze on the nucleus. J Cell Biol. (2016) 215:5–8. 10.1083/jcb.201609102 27697927 PMC5057291

[122] Paul CD Mistriotis P Konstantopoulos K. Cancer cell motility: lessons from migration in confined spaces. Nat Rev Cancer. (2017) 17:131–40. 10.1038/nrc.2016.123 27909339 PMC5364498

[123] Caplan AI. Mesenchymal stem cells: time to change the name! Stem Cells Transl. Med. (2017) 6:1445–51. 10.1002/sctm.17-0051 28452204 PMC5689741

[124] Acosta SA Tajiri N Hoover J Kaneko Y Borlongan CV. Intravenous bone marrow stem cell grafts preferentially migrate to spleen and abrogate chronic inflammation in stroke. Stroke. (2015) 46:2616–27. 10.1161/STROKEAHA.115.009854 26219646 PMC4542567

[125] Laroni A de Rosbo NK Uccelli A. Mesenchymal stem cells for the treatment of neurological diseases: immunoregulation beyond neuroprotection. Immunol Lett. (2015) 168:183–90. 10.1016/j.imlet.2015.08.007 26296458

[126] Grisendi G Spano C Rossignoli F D'Souza N Golinelli G Fiori A. Tumor stroma manipulation by MSC. Curr Drug Targets. (2016) 17:1111–26. 10.2174/1389450117666160307143226 26953248

[127] Karp JM Leng Teo GS. Mesenchymal stem cell homing: the devil is in the details. Cell Stem Cell. (2009) 4:206–16. 10.1016/j.stem.2009.02.001 19265660

[128] Chamberlain G Smith H Rainger GE Middleton J. Mesenchymal stem cells exhibit firm adhesion, crawling, spreading and transmigration across aortic endothelial cells: effects of chemokines and shear. PLoS ONE. (2011) 6:e25663. 10.1371/journal.pone.0025663 21980522 PMC3182247

[129] Cui LL Kerkelä E Bakreen A Nitzsche F Andrzejewska A Nowakowski A. The cerebral embolism evoked by intra-arterial delivery of allogeneic bone marrow mesenchymal stem cells in rats is related to cell dose and infusion velocity. Stem Cell Res Ther. (2015) 6:11. 10.1186/scrt544 25971703 PMC4429328

[130] Teo GS Yang Z Carman CV Karp JM Lin CP. Intravital imaging of mesenchymal stem cell trafficking and association with platelets and neutrophils. Stem Cells. (2015) 33:265–77. 10.1002/stem.1848 25263183 PMC4270897

[131] Nitzsche F Müller C Lukomska B Jolkkonen J Deten A Boltze J. Concise review: MSC adhesion cascade—insights into homing and transendothelial migration. Stem Cells. (2017) 35:1446–60. 10.1002/stem.2614 28316123

[132] Lee H-Y Hong I-S. Double-edged sword of mesenchymal stem cells: cancer-promoting versus therapeutic potential. Cancer Sci. (2017) 108:1939–46. 10.1111/cas.13334 28756624 PMC5623746

[133] Han Y Li XZ Zhang YB Han YP Chang F Ding JX. Mesenchymal stem *cells* for regenerative medicine. Cells. (2019) 8:886. 10.3390/cells8080886 PMC6721852 31412678

[134] Bilir BM Guinette D Karrer F Kumpe DA Krysl J Stephens J. Hepatocyte transplantation in acute liver failure. Liver Transpl. (2000) 6:32–40. 10.1016/S1527-6465(00)80030-1 10648575

[135] Fisher RA Bu D Thompson M Tisnado J Prasad U Sterling R. Defining hepatocellular chimerism in a liver failure patient bridged with hepatocyte infusion. Transplantation. (2000) 69:303–7. 10.1097/00007890-200001270-00018 10670643

[136] Khan AA Habeeb A Parveen N Naseem B Babu RP Capoor AK. Peritoneal transplantation of human fetal hepatocytes for the treatment of acute fatty liver of pregnancy: a case report. Trop Gastroenterol. (2004) 25:141–3. 15682663

[137] Schneider A Attaran M Meier PN Strassburg C Manns MP Ott M. Hepatocyte transplantation in an acute liver failure due to mushroom poisoning. Transplantation. (2006) 82:1115–6. 10.1097/01.tp.0000232451.93703.ab 17060866

[138] Grossman M Rader DJ Muller DW Kolansky DM Kozarsky K Clark BJ III. A pilot study of *ex vivo* gene therapy for homozygous familial hypercholesterolaemia. Nat Med. (1995) 1:1148–54. 10.1038/nm1195-1148 7584986

[139] Dhawan A Mitry RR Hughes RD Lehec S Terry C Bansal S. Hepatocyte transplantation for inherited factor VII deficiency. Transplantation. (2004) 78:1812–4. 10.1097/01.TP.0000146386.77076.47 15614156

[140] Dhawan A Mitry RR Hughes RD. Hepatocyte transplantation for liver-based metabolic disorders. J Inherit Metab Dis. (2006) 29:431–5. 10.1007/s10545-006-0245-8 16763914

[141] Puppi J Tan N Mitry RR Hughes RD Lehec S Mieli-Vergani G. Hepatocyte transplantation followed by auxiliary liver transplantation–a novel treatment for ornithine transcarbamylase deficiency. Am J Transplant. (2008) 8:452–7. 10.1111/j.1600-6143.2007.02058.x 18211511

[142] Asonuma K Gilbert JC Stein JE Takeda T Vacanti JP. Quantitation of transplanted hepatic mass necessary to cure the Gunn rat model of hyperbilirubinemia. J Pediatr Surg. (1992) 27:298–301. 10.1016/0022-3468(92)90850-7 1501000

[143] Jorns C Ellis EC Nowak G Fischler B Nemeth A Strom SC. Hepatocyte transplantation for inherited metabolic diseases of the liver. J Intern Med. (2012) 272:201–23. 10.1111/j.1365-2796.2012.02574.x 22789058

[144] Hansel MC Gramignoli R Skvorak KJ Dorko K Marongiu F Blake W. The history and use of human hepatocytes for the treatment of liver diseases: the first 100 patients. Curr Protoc Toxicol. (2014) 62:14.12.11–23. 10.1002/0471140856.tx1412s62 25378242 PMC4343212

[145] Squires JE Soltys KA McKiernan P Squires RH Strom SC Fox IJ. Clinical hepatocyte transplantation: what is next? Curr Transplant Rep. (2017) 4:280–9. 10.1007/s40472-017-0165-6 29732274 PMC5932623

[146] Gupta S Joseph B. Regulation of cell engraftment in the liver. In: Gupta S Jansen PLM Klempnauer J Manns MP editors. Hepatocyte Transplantation. Netherlands: Springer (2002). p. 15–23.

[147] Gupta S. Hepatocyte transplantation. J Gastroenterol Hepatol. (2002) 17 (Suppl. 3):S287–93. 10.1046/j.1440-1746.17.s3.15.x 12472951

[148] Rajvanshi P Kerr A Bhargava KK Burk RD Gupta S. Studies of liver repopulation using the dipeptidyl peptidase IV-deficient rat and other rodent recipients: cell size and structure relationships regulate capacity for increased transplanted hepatocyte mass in the liver lobule. Hepatology. (1996) 23:482–96. 10.1002/hep.510230313 8617428

[149] Slehria S Rajvanshi P Ito Y Sokhi RP Bhargava KK Palestro CJ. Hepatic sinusoidal vasodilators improve transplanted cell engraftment and ameliorate microcirculatory perturbations in the liver. Hepatology. (2002) 35:1320–8. 10.1053/jhep.2002.33201 12029617

[150] Gustafson EK Elgue G Hughes RD Mitry RR Sanchez J Haglund U. The instant blood-mediated inflammatory reaction characterized in hepatocyte transplantation. Transplantation. (2011) 91:632–8. 10.1097/TP.0b013e31820ae459 21289595

[151] Lee CA Dhawan A Smith RA Mitry RR Fitzpatrick E. Instant blood-mediated inflammatory reaction in hepatocyte transplantation: current status and future perspectives. Cell Transplant. (2016) 25:1227–36. 10.3727/096368916X691286 26996786

[152] Overturf K Al-Dhalimy M Tanguay R Brantly M Ou CN Finegold M. Hepatocytes corrected by gene therapy are selected *in vivo* in a murine model of hereditary tyrosinaemia type I. Nat Genet. (1996) 12:266–73. 10.1038/ng0396-266 8589717

[153] Furrer K Tian Y Pfammatter T Jochum W El-Badry AM Graf R. Selective portal vein embolization and ligation trigger different regenerative responses in the rat liver. Hepatology. (2008) 47:1615–23. 10.1002/hep.22164 18395841

[154] Abdalla EK. Portal vein embolization. (prior to major hepatectomy) effects on regeneration, resectability, and outcome. J Surg Oncol. (2010) 102:960–7. 10.1002/jso.21654 21165999

[155] Gramignoli R Vosough M Kannisto K Srinivasan RC Strom SC. Clinical hepatocyte transplantation: practical limits and possible solutions. Eur Surg Res. (2015) 54:162–77. 10.1159/000369552 25633583

[156] Yamanouchi K Zhou H Roy-Chowdhury N Macaluso F Liu L Yamamoto T. Hepatic irradiation augments engraftment of donor cells following hepatocyte transplantation. Hepatology. (2009) 49:258–67. 10.1002/hep.22573 19003915 PMC3416044

[157] Wang LJ Chen YM George D Smets F Sokal EM Bremer EG. Engraftment assessment in human and mouse liver tissue after sex-mismatched liver cell transplantation by real-time quantitative PCR for Y chromosome sequences. Liver Transpl. (2002) 8:822–8. 10.1053/jlts.2002.34891 12200785

[158] Malhi H Annamaneni P Slehria S Joseph B Bhargava KK Palestro CJ. Cyclophosphamide disrupts hepatic sinusoidal endothelium and improves transplanted cell engraftment in rat liver. Hepatology. (2002) 36:112–21. 10.1053/jhep.2002.33896 12085355

[159] Kumaran V Joseph B Benten D Gupta S. Integrin and extracellular matrix interactions regulate engraftment of transplanted hepatocytes in the rat liver. Gastroenterology. (2005) 129:1643–53. 10.1053/j.gastro.2005.08.006 16285962

[160] Pankowicz FP Barzi M Legras X Hubert L Mi T Tomolonis JA. Reprogramming metabolic pathways *in vivo* with CRISPR/Cas9 genome editing to treat hereditary tyrosinaemia. Nat Commun. (2016) 7:12642. 10.1038/ncomms12642 27572891 PMC5013601

[161] VanLith C Guthman R Nicolas CT Allen K Du Z Joo DJ. Curative *ex vivo* hepatocyte-directed gene editing in a mouse model of hereditary tyrosinemia type 1. Hum Gene Ther. (2018) 29:1315–26. 10.1089/hum.2017.252 29764210 PMC6247987

[162] Krooss SA Dai Z Schmidt F Rovai A Fakhiri J Dhingra A. *Ex vivo*/*in vivo* gene editing in hepatocytes using “all-in-one” CRISPR-adeno-associated virus vectors with a self-linearizing repair template. iScience. (2020) 23:100764. 10.1016/j.isci.2019.100764 31887661 PMC6941859

[163] Nygaard S Barzel A Haft A Major A Finegold M Kay MA. A universal system to select gene-modified hepatocytes *in vivo*. Sci Transl Med. (2016) 8:342ra379. 10.1126/scitranslmed.aad8166 27280686 PMC5242329

[164] Ramaswamy S Tonnu N Menon T Lewis BM Green KT Wampler D. Autologous and heterologous cell therapy for hemophilia B toward functional restoration of factor IX. Cell Rep. (2018) 23:1565–80. 10.1016/j.celrep.2018.03.121 29719266 PMC5987250

[165] Atherton A Born GV. Quantitative investigations of the adhesiveness of circulating polymorphonuclear leucocytes to blood vessel walls. J Physiol. (1972) 222:447–74. 10.1113/jphysiol.1972.sp009808 4624453 PMC1331392

[166] Wong J Johnston B Lee SS Bullard DC Smith CW Beaudet AL. A minimal role for selectins in the recruitment of leukocytes into the inflamed liver microvasculature. J Clin Invest. (1997) 99:2782–90. 10.1172/JCI119468 9169509 PMC508125

[167] Poisson J Lemoinne S Boulanger C Durand F Moreau R Valla D. Liver sinusoidal endothelial cells: physiology and role in liver diseases. J Hepatol. (2017) 66:212–27. 10.1016/j.jhep.2016.07.009 27423426

[168] Frohlich EM Alonso JL Borenstein JT Zhang X Arnaout MA Charest JL. Topographically-patterned porous membranes in a microfluidic device as an *in vitro* model of renal reabsorptive barriers. Lab Chip. (2013) 13:2311–9. 10.1039/c3lc50199j 23636129 PMC4578304

[169] Chung HH Mireles M Kwarta BJ Gaborski TR. Use of porous membranes in tissue barrier and co-culture models. Lab Chip. (2018) 18:1671–89. 10.1039/C7LC01248A 29845145 PMC5997570

[170] Kim MY Li DJ Pham LK Wong BG Hui EE. Microfabrication of high-resolution porous membranes for cell culture. J Memb Sci. (2014) 452:460–9. 10.1016/j.memsci.2013.11.034 24567663 PMC3931465

[171] Allahyari Z Gholizadeh S Chung HH Delgadillo LF Gaborski TR. Micropatterned Poly(ethylene glycol) islands disrupt endothelial cell-substrate interactions differently from microporous membranes. ACS Biomater Sci Eng. (2020) 6:959–68. 10.1021/acsbiomaterials.9b01584 32582838 PMC7314360

[172] Vandenhaute E Drolez A Sevin E Gosselet F Mysiorek C Dehouck MP. Adapting coculture *in vitro* models of the blood-brain barrier for use in cancer research: maintaining an appropriate endothelial monolayer for the assessment of transendothelial migration. Lab Invest. (2016) 96:588–98. 10.1038/labinvest.2016.35 26901835

[173] Carter RN Casillo SM Mazzocchi AR DesOrmeaux JS Roussie JA Gaborski TR. Ultrathin transparent membranes for cellular barrier and co-culture models. Biofabrication. (2017) 9:015019. 10.1088/1758-5090/aa5ba7 28140345 PMC5373106

[174] Gholizadeh S Allahyari Z Carter R Delgadillo L Blaquiere M Nouguier-Morin F. Robust and gradient thickness porous membranes for *in vitro* modeling of physiological barriers. bioRxiv. [Preprint]. (2020). 10.1101/2020.05.07.083188 PMC7942760 33709013

[175] Miller JJ Carter RN McNabb KB Desormeaux JPS Striemer CC Winans JD. Lift-off of large-scale ultrathin nanomembranes. J Micromech Microeng. (2015) 25:15011. 10.1088/0960-1317/25/1/015011

[176] Quirós-Solano WF Gaio N Stassen OMJA Arik YB Silvestri C Van Engeland NCA. Microfabricated tuneable and transferable porous PDMS membranes for organs-on-chips. Sci Rep. (2018) 8:13524. 10.1038/s41598-018-31912-6 30202042 PMC6131253

[177] Agrawal AA Nehilla BJ Reisig KV Gaborski TR Fang DZ Striemer CC. Porous nanocrystalline silicon membranes as highly permeable and molecularly thin substrates for cell culture. Biomaterials. (2010) 31:5408–17. 10.1016/j.biomaterials.2010.03.041 20398927

[178] DesOrmeaux JP Winans JD Wayson SE Gaborski TR Khire TS Striemer CC. Nanoporous silicon nitride membranes fabricated from porous nanocrystalline silicon templates. Nanoscale. (2014) 6:10798–805. 10.1039/C4NR03070B 25105590

[179] Huh D Matthews BD Mammoto A Montoya-Zavala M Hsin HY Ingber DE. Reconstituting organ-level lung functions on a chip. Science. (2010) 328:1662–8. 10.1126/science.1188302 20576885 PMC8335790

[180] Fard AK McKay G Buekenhoudt A Al Sulaiti H Motmans F Khraisheh M. Inorganic membranes: preparation and application for water treatment and desalination. Materials. (2018) 11:74. 10.3390/ma11010074 PMC5793572 29304024

[181] Li J Zhou X Wang J Li X. Two-dimensional covalent organic frameworks. (COFs) for membrane separation: a mini review. Ind Eng Chem Res. (2019) 58:15394–406. 10.1021/acs.iecr.9b02708

